# From intracellular signaling to population oscillations: bridging size- and time-scales in collective behavior

**DOI:** 10.15252/msb.20145352

**Published:** 2015-01-23

**Authors:** Allyson E Sgro, David J Schwab, Javad Noorbakhsh, Troy Mestler, Pankaj Mehta, Thomas Gregor

**Affiliations:** 1 Joseph Henry Laboratories of Physics, Princeton UniversityPrinceton, NJ, USA; 2 Lewis-Sigler Institute for Integrative Genomics, Princeton UniversityPrinceton, NJ, USA; 3 Department of Physics, Boston UniversityBoston, MA, USA

**Keywords:** dynamical systems, FRET, live microscopy, phenomenological modeling

## Abstract

Collective behavior in cellular populations is coordinated by biochemical signaling networks within individual cells. Connecting the dynamics of these intracellular networks to the population phenomena they control poses a considerable challenge because of network complexity and our limited knowledge of kinetic parameters. However, from physical systems, we know that behavioral changes in the individual constituents of a collectively behaving system occur in a limited number of well-defined classes, and these can be described using simple models. Here, we apply such an approach to the emergence of collective oscillations in cellular populations of the social amoeba *Dictyostelium discoideum*. Through direct tests of our model with quantitative *in vivo* measurements of single-cell and population signaling dynamics, we show how a simple model can effectively describe a complex molecular signaling network at multiple size and temporal scales. The model predicts novel noise-driven single-cell and population-level signaling phenomena that we then experimentally observe. Our results suggest that like physical systems, collective behavior in biology may be universal and described using simple mathematical models.

## Introduction

Collective behavior is a common feature of many biological systems and is present in systems ranging from flocking birds, human spectators, schooling fish, and circadian rhythms in many higher organisms, to swarming bacterial colonies, cell migration, and embryonic morphogenesis (Farkas *et al*, 2002; Couzin & Krause, 2003; Waters & Bassler, 2005; Kawano *et al*, 2006; Szabó *et al*, 2006; Ballerini *et al*, 2008; Giardina, 2008; Friedl & Gilmour, 2009; Ullner *et al*, 2009; Zhang *et al*, 2010). In cellular systems that exhibit collective behavior, individual cells must coordinate their behavior with one another to produce the observed population-level phenomena and do so utilizing extracellular small molecules or proteins. For example, bacteria commonly utilize quorum-sensing molecules to synchronize gene expression in cellular populations and form aggregate biofilms (Waters & Bassler, 2005), and synthetic biology has exploited these mechanisms to engineer new circuits that give rise to population-level behaviors (Mondragón-Palomino *et al*, 2011; Youk & Lim, 2014). However, each cell's behavior and its communication with other cells are controlled by complex intracellular biochemical networks. Illuminating how the dynamics of these intracellular networks lead to the population-wide collective behavior observed in these systems is a challenging problem, in part due to the difference in size and temporal scales at which these behaviors are controlled and displayed (Mehta & Gregor, 2010). In this work, we utilize the phenomenon of universality in an effort to connect individual genes to single-cell signaling behaviors and then to relate single-cell activity to population behavior.

A classic example of such collective cellular behaviors is the transition during starvation from an independent, single-celled state to a multicellular aggregate in the eukaryotic social amoeba *Dictyostelium discoideum*. Its population-level behaviors are controlled by a complex biochemical network within individual cells and coordinated through cell–cell communication via the small molecule cyclic AMP (cAMP). During starvation, single cells begin to produce cAMP and up-regulate the expression of signaling network components. Once this signaling machinery is sufficiently expressed, cells can detect the external cAMP and respond by massively producing their own pulse of cAMP internally that is then released into the environment. Released cAMP diffuses through the extracellular environment, relaying the stimulation to other cells and eventually leading to autonomous population-level oscillations (Alcantara & Monk, 1974; Gross *et al*, 1976; Tomchik & Devreotes, 1981; Gregor *et al*, 2010).

Despite our extensive knowledge of the components of the *Dictyostelium* signaling pathway, there is no consensus on how this pathway gives rise to synchronized cAMP oscillations in cellular populations (Martiel & Goldbeter, 1987; Lauzeral *et al*, 1997; Laub & Loomis, 1998; Sawai *et al*, 2005). Not only are there likely other as-yet-undiscovered components in the signaling circuit, the circuit dynamics are poorly understood and these dynamics change with increasing starvation time and changing environmental conditions. As a result, it is challenging to uncover the origins of collective behavior and predict novel behaviors even in a well-studied model organism such as *Dictyostelium* through a detailed, “bottom-up” modeling approach that incorporates each network component and interaction. These challenges are made even more pronounced by the need to bridge multiple timescales. For example, chemotactic responses to cAMP in *Dictyostelium* occur on the order of 30–60 s (Manahan *et al*, 2004; Iglesias & Devreotes, 2008, 2012; Takeda *et al*, 2012; Wang *et al*, 2012), whereas the period of population-level cAMP oscillations are typically an order of magnitude larger (6–10 min) (Tomchik & Devreotes, 1981; Gregor *et al*, 2010). Another approach is clearly needed to elucidate how these complicated single-cell networks give rise to collective population behaviors and to bridge the divide between different temporal and size scales.

Here, we present a general modeling approach for overcoming these challenges based on the concept that population-level behaviors do not depend on all details of the intracellular dynamics of individual members of the population and that a dimensionally reduced system accurately captures the essential phenomena. This approach has been key to understanding collective behavior in physical systems, for example, the equilibrium phase transition from a gas to a liquid (Guckenheimer & Holmes, 1983; Anderson, 1997; Kadanoff, 2000). In dynamical systems, at transitions between two different behaviors, so-called “bifurcations”, only a few qualitatively different behaviors are possible and these can be described by simple, low-dimensional models regardless of the complexity of the system being modeled (Strogatz, 2001; Izhikevich, 2007). This concept is known as “universality” (Kadanoff, 2000).

Many biological systems also undergo bifurcations in their behavior and may thus be amenable to mathematical modeling via a universality-based approach. For example, both isolated *Dictyostelium* cells and cellular populations undergo a bifurcation to oscillations as a function of external cAMP levels (Tomchik & Devreotes, 1981; Gregor *et al*, 2010). Here, we exploit universality to build a simple predictive model of the *Dictyostelium* signaling circuit that reproduces the essential behavior of single cells as well as cellular populations and experimentally confirm its success. This “top-down” modeling approach does not require detailed knowledge of the signaling circuit and is ideally suited for complex biological regulatory networks where kinetic or topological information is limited. Using this approach, we show that a universal model can successfully describe both single-cell and multicellular dynamics in collective biological systems, such as oscillatory cell populations of amoebae or neurons.

## Results

### A 2D-model for *Dictyostelium* signaling dynamics

Population-level *Dictyostelium* signaling dynamics have been experimentally described in great detail (Martiel & Goldbeter, 1987; Laub & Loomis, 1998; Sawai *et al*, 2005; Gregor *et al*, 2010), but a comprehensive model that captures the basic phenomenology and yet retains predictive power is still missing. Thus, guided by experimental observations, our goal is to build a low-dimensional single-cell model, experimentally test its predictions, and then use it as a building block for a model that describes population-level signaling dynamics.

**Figure 1:**
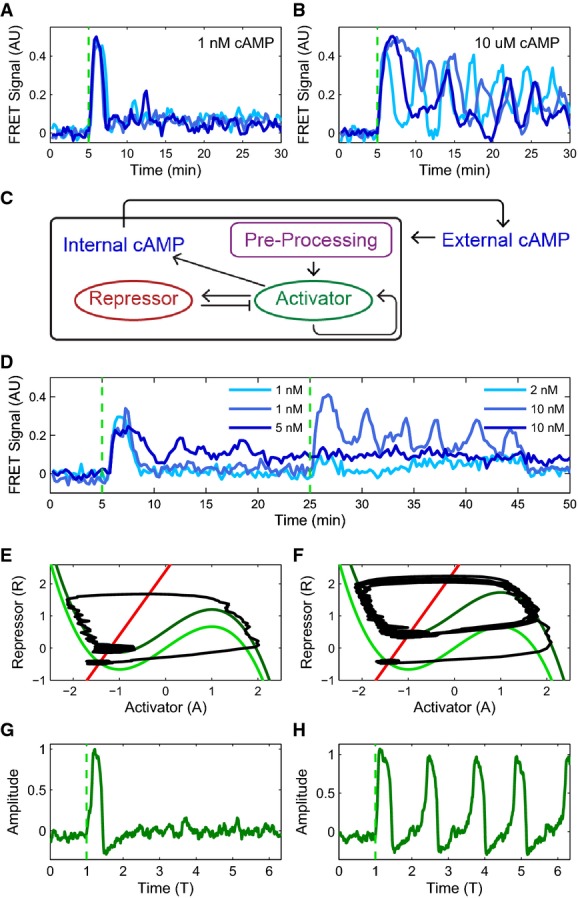
Modeling cytosolic cAMP responses to external cAMP stimuli in individual *Dictyostelium* cells

The key experimental observation underlying our single-cell model is a qualitative change, or bifurcation, in the *Dictyostelium* signaling network's dynamical behavior in response to increasing concentration of extracellular cAMP in a microfluidic device, measured using a FRET sensor (Fig1A and B, Supplementary Figs S1 and S2) (Nikolaev *et al*, 2004; Gregor *et al*, 2010). At low extracellular cAMP levels, cells respond by producing a single pulse of internal cAMP, whereas at high cAMP, cells oscillate. Hence, the extracellular cAMP concentration plays the role of a bifurcation parameter, and the system should be describable by a simple, low-dimensional model (Strogatz, 2001; Izhikevich, 2007). Furthermore, low-dimensional dynamical systems close to a bifurcation can exhibit only a few universal, qualitative behaviors, often termed bifurcation classes. The existence of small, noisy sub-threshold fluctuations in the baseline of internal cAMP levels even in the absence of extracellular cAMP and the fact that varying extracellular cAMP is sufficient for the bifurcation to occur imply that the *Dictyostelium* signaling network is well described by a co-dimension one bifurcation (i.e., only one parameter needs to be varied for the bifurcation to occur), which is the simplest bifurcation class consistent with oscillations. We would therefore like a model that exhibits the following behaviors: an oscillatory bifurcation with no “bistability” between oscillations and silence, finite-frequency oscillations at the bifurcation, and bursts in response to steps below the bifurcation.

Experimental observation of a bifurcation: cytosolic cAMP responses to an externally applied cAMP stimulus of 1 nM (A) and 10 μM (B) at 5 min in three single *Dictyostelium* cells expressing an Epac1camps-FRET sensor (cells stimulated using a custom microfluidics device (see Supplementary Fig S2); FRET signal is a normalized ratiometric fluorescence intensity measurement proportional to cytosolically produced cAMP (Salonikidis *et al*, 2008); see Materials and Methods).

Schematic of proposed model (see text for details).

Cytosolic cAMP responses of single cells in microfluidic devices to successive externally applied cAMP stimuli of 1 nM (light and medium blue) or 5 nM (dark blue) followed by 2 nM (light blue) or 10 nM (medium and dark blue) step.

Phase portraits for a small (E) and a large (F) step stimulus (corresponding to (A) and (B), respectively), with repressor (R) nullcline (i.e., d*R*/d*t* = 0) shown in red and activator (A) nullclines (i.e., d*A*/d*t* = 0) shown in green (see text for model details). For the activator, two nullclines are shown corresponding to a pre-stimulus (light green) and a post-stimulus (dark green) regime. A fixed point for the dynamics occurs where the S-shaped activator nullcline intersects the repressor nullcline (red line). The response trajectory is shown in black. See Supplementary Fig S3 for details about the FHN model fixed point behavior.

Activator variable as a function of time for (G) a small stimulus (corresponding to A, E) and (H) a large stimulus (corresponding to B, F; green dashed line indicates stimulus onset). Data information: For simulated data throughout the paper, time unit “T” is defined as the average minimum period in the FHN model for a single cell, and “Amplitude” is defined as the mean height of spikes at 1 nM “internal cAMP”. Simulated model time courses will be shown in shades of green. Experimental time courses will be shown in shades of blue with time units of min and amplitude in arbitrary FRET signal units. Source data are available online for this figure.

The simplest two-dimensional model that satisfies the above conditions is the excitable FitzHugh–Nagumo (FHN) model (FitzHugh, 1961; Nagumo *et al*, 1962; Izhikevich, 2007; Murray, 2007). The FHN model falls into the supercritical Hopf bifurcation class, where a single stable fixed point transitions into a stable limit cycle that increases in amplitude as the bifurcation parameter increases (Supplementary Fig S3). It has been studied extensively in the theory of dynamical systems and neuroscience (Izhikevich, 2007) and formally has oscillations that rise from zero amplitude and finite frequency with no bistability. A feature of the FHN model is that when its bifurcation parameter is pulled rapidly upward while remaining below the bifurcation threshold, it exhibits a transient spike-like response with a characteristic amplitude, analogous to when neurons produce a voltage spike and fire. Because of that analogy, we adopt these terms from neuroscience to describe cells producing an internal pulse, or spike, of cAMP. Other models of population-level *Dictyostelium* signaling dynamics based on excitability have been proposed (Vasiev *et al*, 1994, 1997; Lee, 1997; Marée *et al*, 1999), but do not capture the details of single-cell behaviors that our model does. Furthermore, while these models and ours are excitable in the sense of displaying large, transient responses to large signal perturbations, these other models do not allow for oscillatory behavior at the single-cell level while extracellular cAMP concentrations are fixed as has been observed experimentally (Gregor *et al*, 2010). The FHN model relies on internal feedback mechanisms to produce this behavior at the single-cell level due to its proximity to an oscillatory bifurcation, as we emphasize below.

The model has two dimensionless dynamical variables: an “activator”, *A*, which activates itself through an auto-regulatory positive feedback, and a “repressor”, *R*, that is activated by *A* and, in turn, inhibits *A* through a slower negative feedback loop (see Fig1C). Mathematically, the noisy FHN is described by the stochastic Langevin equations 

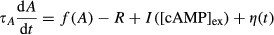
1

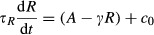
2where the nonlinear function *f*(*A*) = *A* – (1/3)*A*^3^ mimics the effect of a positive feedback loop. The dimensionless parameter *ε = τ*_*A*_/*τ*_*R*_ controls the ratio between the activator and repressor timescale dynamics, that is the excitability; *γ* is the repressor degradation rate, and *c*_0_ sets the steady state repressor value in the absence of external cAMP. The input function *I*([cAMP]_ex_) depends on the experimentally controlled extracellular cAMP concentration, [cAMP]_ex_, and reflects any “pre-processing” modules that may exist upstream of the excitable FHN circuit. Experimentally, we find that the upstream “pre-processing” circuit can sense fold changes in cAMP (Fig1D) and thus is well modeled by *I*(*x*) =*a* log(1 + *x*/*K*_*d*_). *K*_*d*_ corresponds to the threshold for response to cAMP, and *a* determines the magnitude of the response (see SI of Sawai *et al*, 2005). We have also included a Langevin noise term *η*(*t*) that satisfies the relation 〈*η*(*t*) *η*(*t*′)〉 = *σ*^2^*δ*(*t* − *t*′), where *σ*^2^ is a measure of the strength of the noise. Importantly, our qualitative predictions do not depend strongly on the choice of parameters and the form of the nonlinearity of *f*(*A*) (Strogatz, 2001). All we require is that in the absence of external cAMP, the system is below the oscillatory bifurcation and excitable. Nonetheless, parameters were chosen to best fit the experimental data, and a single set of parameters was used throughout this manuscript. Figure1E and F show the phase portraits for a FHN model for externally applied cAMP stimuli (E) below and (F) above the threshold for oscillations, respectively. In the low cAMP phase portrait (Fig1E), the final fixed point is stable and describes the long-time behavior of the system (Fig1G). In contrast, in the high cAMP phase portrait (Fig1F), the fixed point is unstable and the trajectories converge on a limit-cycle attractor for nonlinear oscillations (Fig1H). Thus, the activator concentration *A* is a good proxy for the experimentally observed intracellular cAMP levels, allowing for facile comparison between model and experiments.

One of the prominent behaviors of the FHN model is that in response to steps of external cAMP below the threshold for oscillations (Fig1E), the trajectory makes a long excursion through phase space resulting in a spike of the activator. This excursion produces a transient spike in the “internal cAMP” levels analogous to those seen in experiments (Fig1A). Such spikes have also been observed previously where this behavior was interpreted as “adaptation” of the adenylyl cyclase, ACA, responsible for production of intracellular cAMP in response to changes in extracellular cAMP levels (Comer & Parent, 2006). In contrast, our model here indicates that these so-called “accommodation spikes” result directly from the underlying excitability of the *Dictyostelium* intracellular signaling circuit. Accommodation spikes occur frequently in models of dynamical systems, particularly in the firing of neurons, emphasizing here the connection between these vastly different systems.

### Single *Dictyostelium* cells are excitable feedback systems

Before using this model as a building block for describing cellular populations, we performed a series of experimental tests concentrating on qualitative predictions of our dynamical model that do not depend on the detailed choice of parameters. Our model predictions for the time dependence of activator *A* are well matched to our experimental data for single-cell cytosolic cAMP responses to externally applied cAMP stimuli (Fig1A, B, G, and H). Notice that the model reproduces the initial accommodation spikes for all values of externally applied cAMP followed by oscillations for the 10 μM stimulus. We do find that some longer-term behaviors, such as a slight dampening of the oscillations or down-regulation of noisy firing, do not exactly match our phenomenological model as it is lacking additional terms that would represent such long-term behavior. We suspect that this is due to genetic regulation becoming a factor in our experiments at these longer (>10 min) timescales as *Dictyostelium* is known to regulate gene expression based on cAMP exposure and stimulus shape (Mann & Firtel, 1989). While adaptation processes are clearly at work over longer timescales, in this work, we focus only on the shorter time dynamics in an effort to understand the dynamical mechanisms underlying the signal relay response for a given adaptation state. However, cells typically never experience such uniformly elevated levels of external cAMP as applied here, and our model correctly reproduces single-cell responses to all naturalistic stimuli.

**Figure 2:**
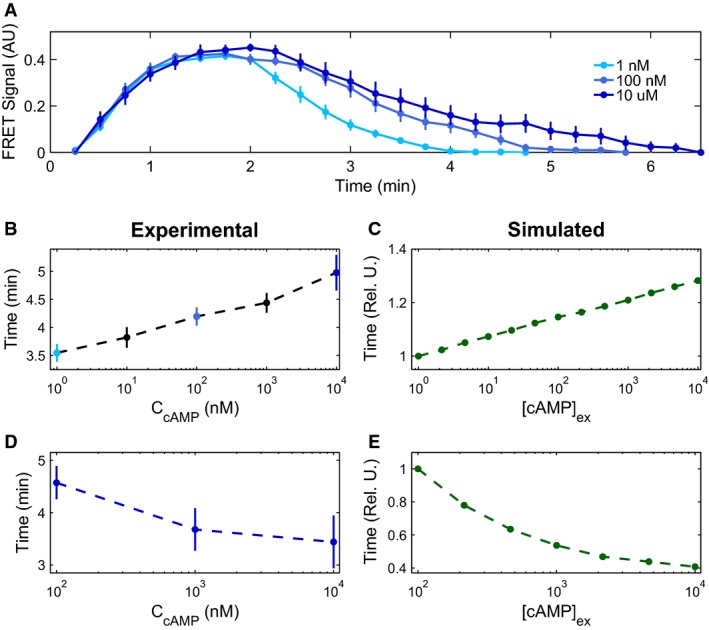
Phenomenological agreement between model and experiments

Over a wide range of cAMP concentrations in experiments, accommodation spikes quickly increase to their peak value, but differ in their decay time back to baseline (Fig2A). Our model predicted and we subsequently experimentally verified that accommodation spike widths monotonically increase with increasing extracellular cAMP concentration (Fig2B and C), but the period of the ensuing oscillations for stimuli of 100 nM cAMP and above decreases as extracellular cAMP concentration increases (Fig2D and E). Together, these results confirm that our model accurately represents the internal cAMP dynamics governing both the initial accommodation spike and the subsequent oscillatory behavior, suggesting that the same molecular mechanism underlies both phenomena. This demonstrates that although single-cell oscillations are not observed in natural conditions, we can make predictions about natural behaviors from modeling their existence. Note that the observed scaling of mean accommodation spike widths scales logarithmically with increasing cAMP (Fig2B), further validating our choice of a logarithmic pre-processing module. Furthermore, the spike width scaling is also inconsistent with the dynamics of adaptation by an incoherent feedforward network such as those proposed to govern *Dictyostelium* chemotaxis (Takeda *et al*, 2012; Wang *et al*, 2012). These results confirm that the full cAMP signaling circuit is best described using an intracellular feedback mechanism, and provide evidence that the observed dynamics result from an underlying negative feedback architecture.

Experimental mean accommodation spikes of cells in microfluidic devices for externally applied cAMP stimuli of 1 nM (light blue), 100 nM (medium blue), and 10 μM (dark blue) (see main text for discussion). Error bars represent SEM.

Experimental (*n* = 16, 14, 14, 20, and 11 cells) (B) and modeled (C) mean initial accommodation spike widths. Error bars represent SEM. Colored data points in (B) correspond to data in (A), with additional mean accommodation spike widths taken at 10 nM and 1 μM.

Experimental (*n* = 11, 16, 10 cells) (D) and modeled (E) mean oscillation times, with experimental mean oscillations found by identifying the peak Fourier transform. Error bars represent errors by bootstrapping. Source data are available online for this figure.

### Single cells are sensitive to the rate of stimulus change, not threshold sensors

**Figure 3:**
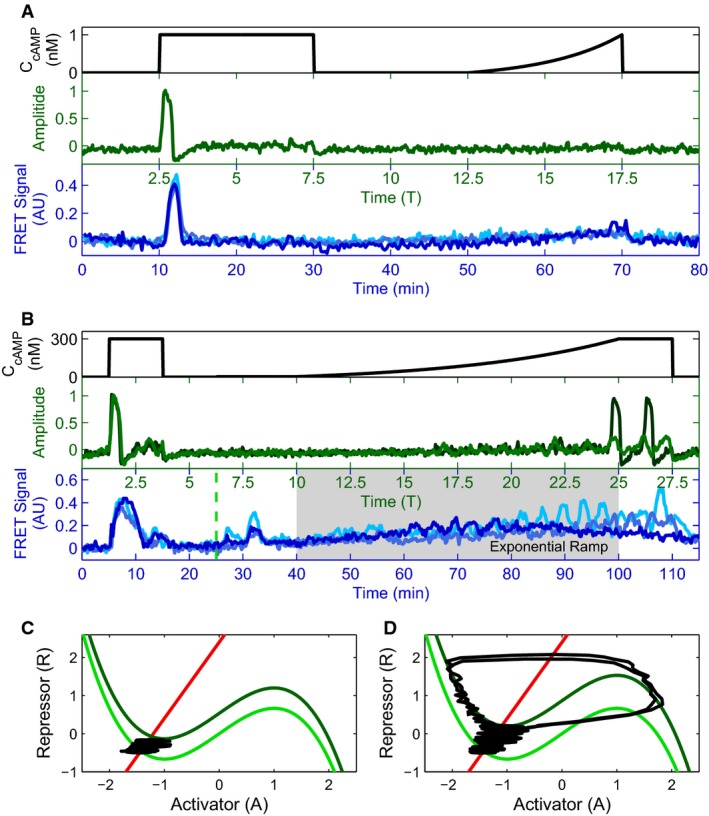
Cytosolic cAMP responses depend on the rate of externally applied cAMP

One of the most interesting predictions of our model is that internal cAMP responses can depend on the rate of externally applied cAMP levels (Fig3A and B) (Levine *et al*, 1996; Sawai *et al*, 2005). In particular, the model predicts that the signal propagation circuit will respond with an accommodation spike in response to a sub-oscillation threshold step of external cAMP (e.g., 1 nM), but will show no response for a slow ramp to the same external cAMP level (Fig3A). The underlying reason for this difference in responses to a step or fast ramp vs. a slow ramp is best understood through the phase portrait (Fig3A and C). For a sufficiently slow ramp of stimulus, the dynamics of the system can follow the stable fixed point as it moves through phase space, without ever leaving its equilibrated state, analogously to a thermodynamic system that follows a slow temperature change adiabatically. In contrast, for fast changes in external cAMP levels, the dynamics are no longer adiabatic and the large sudden change in position of the stable fixed point elicits an accommodation spike. Our experimental results are in agreement with our simulated model predictions (Fig3A), showing that cells can be sensitive to the rate of change of stimulus, and are in direct contrast to previous model assumptions that single cells behave as binary threshold sensors that spike as soon as a certain extracellular cAMP concentration is achieved (Levine *et al*, 1996; Sawai *et al*, 2005). Similar behavior was recently experimentally observed in the stress response of *Bacillus subtilis* (Young *et al*, 2013).

Externally applied cAMP stimuli (black) with a step and an exponential ramp to a final height of 1 nM cAMP (A), and with a small intermediate step (3 nM height, ranging from green dashed line at 25–40 min to give system time to equilibrate), and an exponential ramp to a final height of 300 nM cAMP (time of experimental ramp denoted with gray background, ranging from time 40–100 min) (B). Corresponding activator variable (green) and experimental data for three cells in microfluidic devices (blues) as a function of time are shown below. Model traces are shown for two different degrees of cellular excitability, *∈* = 0.1 (light green) and 0.2 (dark green), showing a diversity of responses similar to that seen experimentally.

Phase portraits for a small (C) and a large (D) exponential ramp stimulus (corresponding to (A) and (B), respectively); R nullcline shown in red, pre-stimulus A nullcline shown in light green, and post-stimulus A nullcline in dark green. The response trajectory is shown in black. Source data are available online for this figure.

A second test of our model is the response of the *Dictyostelium* signal propagation circuit to a large exponential ramp of externally applied cAMP that transitions the system from a sub- to a super-oscillation threshold level (e.g., 100 pM to 300 nM). Our model predicts that once the external cAMP levels are increased adiabatically beyond a critical threshold value where the fixed point changes stability, the system will start oscillating with the amplitude of the oscillations growing with increasing externally applied cAMP (Fig3B and D). Although we are limited both experimentally through the use of syringe pumps and by the time window of the developmental phase we are examining in our ability to probe different ramping speeds and heights that may affect the generalizability of this result, we again find that single cells can be sensitive to the rate of change of stimulus as our model predicts (Fig3B). This behavior validates our initial assumptions that the *Dictyostelium* intracellular signaling circuit is in the supercritical Hopf bifurcation class and can be described by the FHN model (Strogatz, 2001). While the timescales involved in these ramp experiments are long, cells remain quiescent and do not respond to cAMP stimulus with production of their own cytosolic cAMP during the majority of the experiment. Thus we speculate that the “activity-dependent” gene regulation that causes our model predictions to diverge from experimentally observed behaviors during long, constant exposure to cAMP (e.g., oscillation dampening) is not an issue in these experiments. Our suggestion of activity-dependent adaptation is only one possible explanation of the slow ramp behavior, and we leave a detailed investigation to future work.

### Single-cell properties bound multicellular behaviors

**Figure 4:**
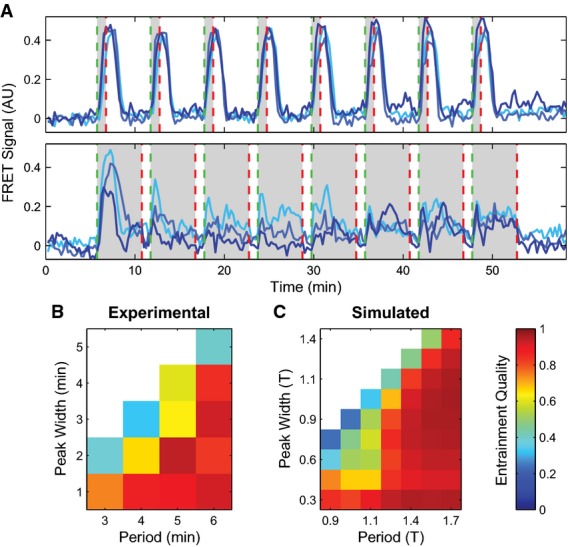
Cytosolic cAMP responses are entrainable to external cAMP stimuli and have a refractory period

As a final test of our single-cell model, we probed how single cells respond to repetitive stimulation of the type seen during collective oscillations. While excitable systems respond to small changes in inputs, they also have a large refractory period where they become insensitive to further stimulation after the stimulus is withdrawn, not merely after its original onset. This interplay between the ability to respond to pulses, immediately followed by a refractory period, can be probed by subjecting cells to pulses with different widths and subsequent rest periods. Experimentally, single cells can easily be entrained to short (1 min) pulses of external cAMP with a long (5 min) rest period between each pulse. However, when the pulse width is increased to 5 min with only a minute of rest between each pulse, poor entrainment results (Fig4A). We quantified single-cell responses to a range of pulse widths and pulse periods, measuring entrainment quality as the mean correlation between the first period response and subsequent period responses (Fig4B). As pulse widths approach the full length of the period, there is insufficient rest time for the system to relax to its previous steady state and thus poor entrainment results. Similarly, our model simulations produce a well-entrained response to a variety of pulse widths, but only after a sufficient refractory period has passed (Fig4C).

Cytosolic cAMP responses of single cells in microfluidic devices to externally applied 10 nM cAMP pulses of 1 min (top) and 5 min (bottom) with a 6-min period (green dashed lines indicate stimulus onsets; red dashed lines indicate stimulus conclusions).

Phase diagrams summarizing 63 single-cell experimental (B) and simulated (C) responses to various pulse widths and periods. “Entrainment Quality” is the mean Pearson correlation coefficient between the first period response and subsequent responses and is represented in color. Red regions display high correlation, while blue regions have low correlation. Source data are available online for this figure.

Entrainment is a natural experimental test of the *Dictyostelium* intracellular signaling network. During the aggregation stage of development, cells detect waves of external cAMP similar to the pulses in our entrainment experiments. Our experiments indicate that a pulse of 3–4 min must be followed by a 2–3-min refractory period for entrainment to occur. These observations are consistent with estimates of the cAMP wave widths and periods found in aggregating populations (Tomchik & Devreotes, 1981). Furthermore, as shown in Fig2A, the 3–4-min pulse width seen in aggregating populations can naturally arise from single-cell accommodation spikes in response to a wide range of external cAMP inputs. Together, these results suggest that the excitability of individual cells places strong limitations on the dynamics of collective oscillations in *Dictyostelium* populations.

### Population model reproduces critical behaviors

Thus far, we have theoretically reproduced a wide range of single-cell behaviors. However, to extend our mathematical description to cellular populations and their autonomous, synchronized oscillations, an explicit model for the dynamics of extracellular cAMP is necessary. We capture these dynamics by a simple mean-field approach, meaning that cells and stimulant are well mixed as they are initially in experiments, thus neglecting spatial detail that arises as cells self-organize at longer timescales. Our multi-cell model builds on the basic structure of the adapted FHN model above where each cell *i* is described by an activator *A*_*i*_ and a repressor *R*_*i*_ (Equations 1 and 2, respectively). Cells secrete low levels of cAMP at a constant baseline rate *α*_0_, and extracellular cAMP is degraded at a rate *D*. In addition, when a cell spikes, it releases a large cAMP pulse into the environment at a rate *S*. Finally, experimentally we can also flow additional cAMP into the system at a concentration *α*_*f*_. These behaviors are captured by a system of Langevin equations of the form 

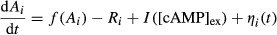
3

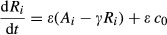
4

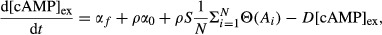
5where *η*_*i*_(*t*) is a cell-dependent Gaussian white noise term with 〈*n*_*i*_(*t*) *n*_*j*_(*t*′)〉 = *σ*^2^*δ*_*ij*_*δ*(*t* − *t*′), and *ρ* is the cell density by volume. Equation 5 describes the spike-driven secretion of cAMP into the medium by the sum, 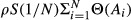 with Θ(*A*_*i*_) being the Heaviside function, which is equal to 1 if *A*_*i*_ > 0 and 0 otherwise. Note that the degradation rate *D* = *J* + *α*_*PDE*_ *ρ*, where *α*_*PDE*_ is the basal rate of phosphodiesterase secretion, can be modulated in our experimental setup by changing the flow rate *J* in our microfluidic devices (see Materials and Methods).

**Figure 5:**
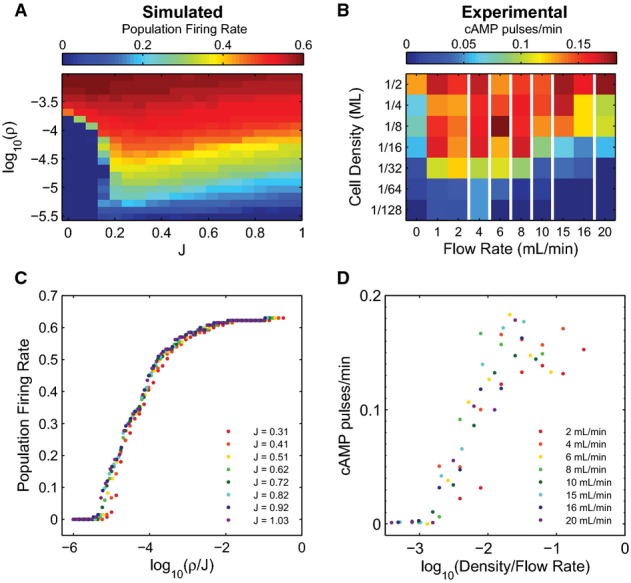
Multicellular model reproduces population behaviors in varying extracellular environments

Our model reproduces the phase diagram for a wide range of environmental conditions from Gregor *et al* (2010), indicating that our mean-field approach is sufficient to describe the autonomous, synchronized population-level oscillations exhibited in *Dictyostelium* populations (Fig5A and B). Note that these collective oscillations are actually synchronous accommodation spikes, not the induced oscillations resulting from an unstable limit cycle as with the single-cell oscillations. Plotting the population firing rate in terms of *ρ*/*J* results in a data collapse for sufficiently large J, and we find good agreement between the model-predicted firing rate dependence on *ρ*/*J* (Fig5C) and the previously published data (replotted in Fig5D). The ability of our model to accurately reproduce observed population-level behaviors over a wide range of experimental conditions demonstrates the success of our strategy of using the carefully calibrated single-cell model as a building block for a multi-cell description.

A phase diagram showing the coordinated population firing rate spanning a range of cell densities and flow rates for the model (A) and experiments in macrofluidic dishes from Gregor *et al* (2010) (B), with the mean firing rate represented in color, and white vertical lines indicate nonlinear breaks in the *x*-axis.

Firing rates can also be considered as a function of the ratio between cell density and flow rate, *ρ*/*J*, as predicted by the model (C) and shown experimentally using macrofluidic dishes in Gregor *et al* (2010) (D). Low flow rates are not plotted in (D) because in this regime, the effect of extracellular PDE is non-negligible. Model firing rates are normalized to an arbitrarily high frequency (˜1/30) to scale maximum values to 1. Source data are available online for this figure.

**Figure 6:**
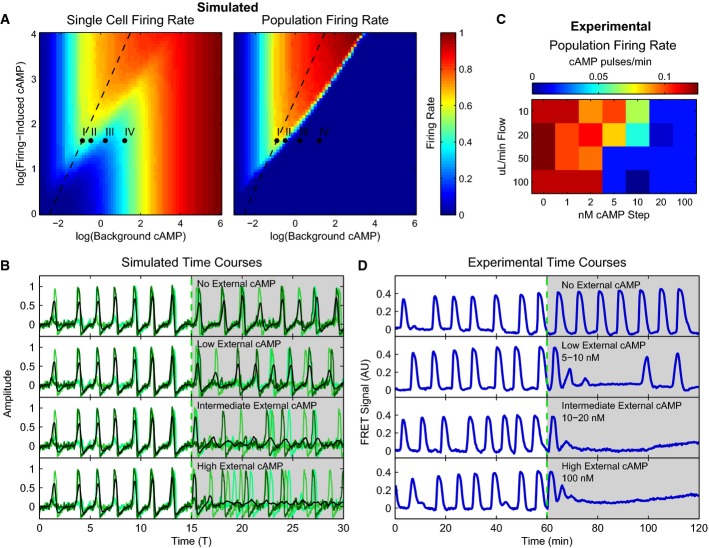
Population model predicts slowing and decoupling of intracellular cAMP oscillations in a population with increased external cAMP

When the flow rate *J* is sufficiently high, the external cAMP concentration dynamics become fast, leading to a separation of timescales between individual cell dynamics and external cAMP dynamics. As a result, external cAMP can be thought of as quickly reaching a quasi-steady state. This assumption dramatically simplifies our analysis of the model, because it allows us to ignore the dynamics of the external medium. Conceptually, it is helpful to think of the extracellular cAMP as originating through two distinct processes: the “firing-induced cAMP”, *ρS*/*J*, which measures the cAMP released by cells when they spike, and the “background cAMP”, (*α*_*f*_ + *ρα*_0_)/*J*, which is the cAMP present even when cells do not spike. Together, these two sources of cAMP are sufficient to describe the external medium and constitute the basis variables for computing single-cell and population firing rates (Fig6A). The single-cell firing rate is a measure of how often an individual cell in the population fires, averaged over the population; the population firing rate is a measure of how often the population fires synchronously. In a coherent population, these measures produce similar results, whereas in an incoherent population, the population firing rate vanishes. Together, these two quantities provide a succinct way to summarize the behavior of the system and allow us to determine in which areas of phase space cells are oscillating and whether these oscillations are synchronous or asynchronous.

Firing rate phase diagrams for single cells in a population and the population as a whole as predicted by our model as a function of background and firing-induced cAMP.

Average “internal cAMP” responses of single simulated cells within a population (greens) and the population mean (black) responses at Points I–IV in (A) as simulated by the model.

Firing rate of 43 experimental populations in microfluidic devices with increasing external cAMP as a function of flow rate for cells plated at >0.5 ML (1 ML = 6,600 cells/mm^2^), imaged 2 mm from population edge. All spikes at least 0.3 FRET signal units in height.

Experimental population average cytosolic cAMP levels for experiments in microfluidic devices with 10 μl/min flow for no externally applied cAMP, 10 nM (low), 20 nM (intermediate), and 100 nM (high) steps of externally applied cAMP. Depending on fluid flow rates and externally applied cAMP levels, populations oscillate, have slow synchronous oscillations, do not oscillate but randomly fire, or oscillate asynchronously (stimulus onset for all assays is at 60 min). Source data are available online for this figure.

To probe the predictive power of our population-level model, we performed a series of simulations where collectively oscillating cellular populations are subject to step stimuli of additional external cAMP into the medium (Fig6B). Considering the phase diagrams in Fig6A, the addition of extracellular cAMP increases the parameter *α*_*f*_ and hence moves the system “horizontally” in the phase diagram, in contrast to changing the cell density or external flow rate, which moves the system “diagonally” through the phase diagram. Without direct access to extracellular cAMP concentrations, *a priori* it is unclear where natural populations are located in this phase diagram. One possibility is that to minimize energy expenditure while still allowing for synchronization, collectively oscillating *Dictyostelium* populations reside in “knee region” of Fig6A labeled Point I. If so, flowing in a small amount of additional extracellular cAMP to these populations will lead to slowing of the synchronized population-level oscillations (Point II in the phase diagram), and a further increase in the amount of extracellular cAMP will result in a loss of oscillations both at the level of single cells and cellular populations (Point III). These behaviors are in stark contrast to that of single cells where flowing in additional cAMP always increases the firing rate. The model also suggests that adding even more external cAMP to the system (Point IV) will cause individual cells to start oscillating asynchronously. In this regime, individuals have a high firing rate but fire incoherently resulting in a negligible populating firing rate.

To test these predictions, we probed these autonomously oscillating populations of *Dictyostelium* with varying levels of additional cAMP at multiple flow rates (see Fig6C and D). For extremely low levels of added extracellular cAMP, the cellular population continues to oscillate synchronously. However, when we increase the cAMP levels further (∽2–10 nM), the oscillations slow down. Eventually, collective oscillations disappear for intermediate levels of cAMP (10–20 nM). Finally, when the extracellular cAMP concentration is increased to extremely high levels (≥100 nM), there is a marked increase in baseline level of the population-level FRET signal, indicating unsynchronized, autonomous oscillations of single cells. The ability to continue to synchronize oscillations over low background levels of cAMP (∽2–10 nM) allows populations to maintain collective states in environments where degradation of secreted cAMP is never complete.

### Intracellular noise drives population-level phenomena

**Figure 7:**
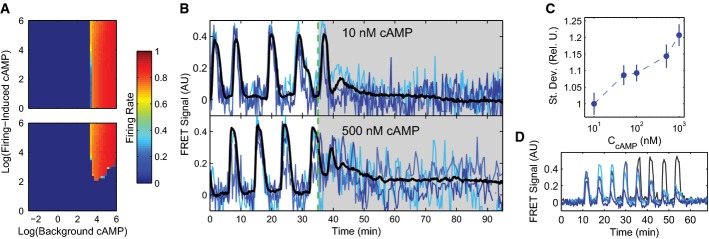
Intracellular noise in the cAMP circuit drives observed population behaviors

The agreement between our simulated phase diagram and population-level experiments in Figs5 and 6 suggests that our phenomenological model correctly captures the key biological mechanisms that give rise to collective oscillations in *Dictyostelium* populations. However, the question of what are the key biological mechanisms beyond a positive feedback loop remains unanswered. Previous work on autonomously oscillating *Dictyostelium* populations strongly suggests noise may also be a key to the onset of collective oscillations (Gregor *et al*, 2010). To elucidate the role of noise in the intracellular cAMP circuit in the emergence of these collective oscillations, we recomputed our phase diagram from Fig6A for various levels and sources of noise in equation 3 (see Supplementary Fig S4). In the absence of stochasticity (Fig7A and Supplementary Fig S4B) or for noise solely in the external cAMP levels (Supplementary Fig S4C), the phase diagram loses almost all of the structure seen in Fig6A. Specifically, the knee region where synchronously oscillating *Dictyostelium* naturally reside without additional external cAMP (i.e., Point I) disappears in the single-cell phase diagram, and the simulation results are inconsistent with the data collapse seen in Fig5. However, the firing rate of individual cells near the oscillatory bifurcation depends strongly on noise, and population-level oscillations emerge in our model when one or a few cells stochastically spike and drive the rest of the population into synchrony. Therefore, intracellular noise introduces a form of “stochastic heterogeneity” among identical cells that drives collective synchronization in a mechanism that resembles coherence resonance, a well-characterized phenomenon in the FHN and similar models, where noise drives the stochastic oscillations in an excitable system into coherence (Wang *et al*, 2000; Lindner *et al*, 2004; DeVille *et al*, 2005; Kitajima & Kurths, 2005). To test whether similar effects could also arise from cell-to-cell heterogeneity in the levels of extracellular cAMP needed to induce a cytosolic spike, we repeated the simulations with a mixture of cells drawn from a lognormal distribution of *K*_*d*_. We found that the resulting phase diagram again has a different shape than for a homogenous, noisy population and is insufficient to reproduce the observed population behaviors (Supplementary Fig S4D). These simulations indicate that in the presence of small amounts of extracellular cAMP, heterogeneity alone cannot drive synchronized population oscillations.

Firing rate phase diagrams for single cells in a population (top) and the population as a whole (bottom) as predicted by the model with minimal noise (*σ *= 0.01) as a function of background and firing-induced cAMP. See Supplementary Fig S4 for other noise-source cases.

Example single-cell (blues) and population (black) cytosolic cAMP traces taken from dual-expressing Epac1camps/mRFPmars tracer cells for oscillating populations at ˜0.4 ML density, 10 μl/min flow subjected to steps of 10 nM and 500 nM cAMP in microfluidic devices.

Mean standard deviations of the single-cell cytosolic cAMP levels for cells in 10 experimental populations inside microfluidic devices subjected to a step stimulus of cAMP as shown in (B) from 10 to 60 min post-stimulus. Values are normalized to the mean standard deviation of cells exposed to a 10 nM external cAMP step to show the relative increase in stochastic variability; errors by bootstrapping.

Single-cell cytosolic cAMP responses to eight 1-nM pulses, 6-min period with 1-min-long pulses (gray) and two 1-min, two 30-s, two 20-s, and two 10-s pulses (blues) given using microfluidic devices. Source data are available online for this figure.

In the simulations, for regimes with intermediate and high extracellular cAMP levels, synchronized population spikes no longer occur. In these regimes, our model predicts that stochasticity drives unsynchronized spiking and that that spiking increases with increasing external cAMP levels (Fig6A and B). Testing this prediction experimentally lets us verify directly whether intracellular noise indeed drives the observed population dynamics. To observe the presence of unsynchronized spiking, we measured cytosolic cAMP levels over time for single cells in autonomously oscillating populations that were subjected to external cAMP steps (Fig7B). We find that in populations the standard deviation of single-cell cytosolic cAMP levels increases with increasing external cAMP levels (Fig7C). This roughly 21% increase in the standard deviation over time in conjunction with the increased population mean cAMP levels indicates that the single-cell levels of cytosolic cAMP themselves are noisier with increased spike height and/or frequency as external cAMP levels increase. While few clear accommodation spike-like cAMP peaks appear to be present after external cAMP is applied (Fig7B), noise-driven spiking may not always result in full-size cAMP peaks like those that result from natural cAMP waves or step stimuli. For example, while a 1-min 1-nM external stimulus is sufficient to produce a full-size peak of internal cAMP in a single cell such as those shown in Figs1, 2, 3 and 4, external cAMP stimuli that are shorter than 20 s produce smaller peaks of internal cAMP (Fig7D) that are qualitatively similar to those seen in Fig7B. This behavior in response to short stimuli is consistent with our model. Together, our model and experiments suggest that *Dictyostelium* populations not only exploit stochasticity in their underlying intracellular signaling network to initiate collective population-level behaviors, but also to coordinate them in noisy extracellular environments.

## Discussion

Through taking advantage of experimental advances that allow for quantitative measurements of intracellular cAMP dynamics in response to a wide variety of extracellular cAMP stimuli and environmental conditions, we have developed a new conceptual framework for understanding collective behavior in cellular populations. Our framework is based on the phenomenon of universality, and we have used it for analyzing the emergence of collective oscillations in the social amoebae *Dictyostelium discoideum*. Our approach allows us to accurately predict and reproduce a wide variety of experimentally confirmed complex dynamical behaviors at both the single-cell and population levels despite having minimal knowledge about the kinetic parameters and interactions of the underlying circuit. Together, our experiments and model revealed that the dynamics of the *Dictyostelium* intracellular signaling network can be understood using a simple two-variable model, specifically the noisy and excitable FitzHugh-Nagumo model. We experimentally showed that individual cells can be sensitive to the dynamics of the input signal and respond differently to steps and ramps of extracellular cAMP. We also experimentally showed that the excitability of individual cells leads to entrainment properties that fundamentally constrain the dynamics of population-level oscillations. When we extend our model to cellular populations, synchronized oscillations spontaneously arise from stochastic accommodation spikes, suggesting that *Dictyostelium* cells actively exploit stochasticity in the biochemical network for controlling population-level behaviors.

Our simple model explains a number of disparate biological phenomena observed during the initiation of the collective phase of the *Dictyostelium* life cycle. For example, it has been shown that when subjected to steps of external cAMP, adenyl cyclase A, which is responsible for cAMP synthesis, shows an initial peak of activation followed by a period in which its activity subsides even in the presence of stimulus (Comer & Parent, 2006). We have shown that this behavior naturally arises from the excitability of the *Dictyostelium* intracellular signaling circuit. Furthermore, it was shown that mutants lacking phosphoinositide 3-kinases PI3K1 and PI3K2 no longer exhibit this accommodation spike behavior and continually produce cAMP (Comer & Parent, 2006). The data from these experiments are consistent with the idea that the mutants undergo a bifurcation to oscillation at lower levels of extracellular cAMP. Our model also suggests a natural explanation for why *Dictyostelium* produce extracellular phosphodiesterases (PDEs) to degrade extracellular cAMP (reviewed in Saran *et al*, 2002). In the absence of degradation, the dynamics of the extracellular cAMP can no longer track the intracellular dynamics of cells, resulting in a loss of coherent oscillations. This loss of coherence is a generic phenomenon present in oscillator systems that communicate through an external medium (Schwab *et al*, 2012a,b). This is consistent with experiments on mutants showing that cells lacking extracellular PDEs do not give rise to spiral waves (Sawai *et al*, 2007) as well as the loss of population oscillations in the presence of the PDE inhibitor DTT (Gregor *et al*, 2010).

In addition to resolving these disparities in our understanding of *Dictyostelium* intracellular signaling dynamics, our model also allows us to discriminate between possible signaling network architectures. Currently, there is no consensus on the architecture of the *Dictyostelium* signaling circuit downstream of the CAR1 receptors (Laub & Loomis, 1998; Kimmel & Parent, 2003; Sawai *et al*, 2005; Bagorda *et al*, 2009; Takeda *et al*, 2012; Wang *et al*, 2012; Nakajima *et al*, 2014; Skoge *et al*, 2014). Our results indicate that there must be a negative feedback loop that turns off production of intracellular cAMP and that there is an as-yet-undiscovered intracellular positive feedback in the circuit, represented in our model by *f*(*A*) = *A* − (1/3)*A*^3^. In contrast with recent work showing that upstream of cAMP production, the *Dictyostelium* chemotaxis network and its output are well described by a feedforward network architecture (Takeda *et al*, 2012; Wang *et al*, 2012; Nakajima *et al*, 2014; Skoge *et al*, 2014), our cAMP signaling circuit uses a network architecture based on feedback. These incoherent feedforward loops cannot exhibit intracellular cAMP oscillations such as those we observe at higher external cAMP concentrations in single cells, making them incompatible with our observations of the signal relay network. Thus, the *Dictyostelium* chemotaxis and signal propagation networks differ in their underlying functional topologies (feedforward versus feedback), even though they likely have shared components and interactions. Indeed, this feedforward network may be the source of the logarithmic pre-processing we observe. One reason for this discrepancy is that the chemotaxis and signal propagation networks operate at fundamentally different timescales. Whereas the gradient sensing response is measured to be on the order of 30 s (Manahan *et al*, 2004; Iglesias & Devreotes, 2008, 2012), the signaling network response operates on a timescale that is nearly an order of magnitude larger (Tomchik & Devreotes, 1981; Gregor *et al*, 2010). For example, the chemotactic response of the pleckstrin homology (PH) domain of cytosolic regulator of adenylyl cyclase (CRAC) dynamics occurs in tens of seconds and not in minutes as is the case of internal cAMP (Wang *et al*, 2012). This difference in timescales likely reflects different biological functions: The chemotaxis network is designed primarily to climb shallow gradients, whereas the signal propagation network is designed to allow cells to aggregate into multicellular structures.

While FHN-inspired models have been previously used to describe the spatial features of population aggregation and cAMP waves in aggregates and slugs (Vasiev *et al*, 1994, 1997; Lee, 1997; Marée *et al*, 1999), they neglect the intracellular signaling dynamics of the signaling relay circuit and that are critical to our results. Furthermore, the excitable mechanism underlying collective oscillations in our coupled FHN population model differs fundamentally in several ways from earlier mathematical models for collective oscillations in *Dictyostelium*. First, classical models of *Dictyostelium* oscillations posited that cells re-sense their own secreted cAMP and that this was the mechanism through which oscillations emerged (Martiel & Goldbeter, 1987). Our experimental results demonstrate that there is an intracellular, not extracellular, feedback look that causes cells to oscillate when external cAMP concentrations are fixed at a high level. Second, previous models treated cells as threshold sensors that emit a pulse of cAMP whenever the extracellular concentration of cAMP passed a threshold (Levine *et al*, 1996; Sawai *et al*, 2005). Our model is similar in spirit to integrate-and-fire models in neuroscience, with cells only spiking once their internal state reaches a threshold. While these input-threshold models are successful at describing aspects of population-level behavior, particularly spatial features, they are inconsistent with the dynamics we observe at the single-cell level (Fig3A). All of these models are excitable in the sense that they display large, transient responses to large external perturbations. However, our implementation of the FNH model at the single-cell level generates these responses through proximity to an oscillatory bifurcation and is unique among these models in that it is driven by internal, positive feedback.

We emphasize that we have neglected several phenomena in crafting our simple model and that these simplifications leave open the possibility that there may be alternative models that also explain our single-cell and population-level data. It is likely that due to changes in gene expression during development, many molecular components may vary on the timescale of hours (Mann & Firtel, 1989). In our model, this could manifest in a number of ways, including changes in the value of parameters with time as well as minor changes in the shape of the corresponding nullclines. We also have ignored the dynamics of adaptation mechanisms and modules that lie upstream of our excitable circuit. This is one possible reason why our model does not reproduce the damped oscillations observed in response to prolonged stimuli of cAMP. However, it is worth emphasizing that there may be other equally plausible explanations for these experimentally observed behaviors. Our model also does not distinguish between the activator variable, A, and the internal levels of cAMP produced when the activator is spiking. This distinction may be important for understanding certain phenomena such as adaptation. Finally, we note that the FHN model does not reproduce the experimentally observed spike shape and that the experimentally observed spike shape does not always match the average, stereotyped response, likely due to cells that do not remain flat on the surface of the coverslip when becoming rounded upon stimulation with cAMP (Alcantara & Monk, 1974). While the model could be modified to agree with each experimentally observed detail by introducing additional fitting parameters, this would significantly complicate the model and limit its power in predicting phenomena. Since our model predictions do not depend on spike shape but general phenomenology, we chose not to do this here. Nonetheless, our experimental results suggest that these alternative models will share certain basic fundamental features with our FHN-based model, including a core negative feedback loop that gives rise to oscillations and stochasticity.

The work presented here suggests that *Dictyostelium* cells actively exploit stochasticity in the signal propagation network to initiate and control population-level oscillations, and while more examples of this type of exploitation are coming to light, it is still clear that in many biological systems, noise limits or degrades biological function (Eldar & Elowitz, 2010; Pilpel, 2011; Sanchez *et al*, 2013). Specifically, the increased likelihood of stochastic spiking at higher extracellular cAMP levels suggests a possible mechanism behind the origin of autonomous oscillation centers. The slow leakage of cAMP during development postulated by Gregor *et al* (2010) will likely not induce accommodation spikes directly as cells can be sensitive to the rate of extracellular cAMP change (Fig3A), but the extracellular cAMP that builds up will encourage increased stochastic spiking. These initial stochastic spikes release comparatively large amounts of cAMP into the extracellular environment, triggering other cells to spike and driving the cells that fired first to become the origins of autonomous oscillation centers. This same stochasticity in the intracellular signaling network drives cells to fire when background levels of cAMP do not continually reset to zero, allowing for continued collective oscillations at times when complete degradation of extracellular cAMP does not occur.

Our work offers a bridge between the disparate fields of collective cellular signaling and neuroscience. The FHN model successfully describes the dynamics of both neurons and *Dictyostelium* cells. Neurons use electrical impulses on the order of a millisecond to communicate directly via synapses. In contrast, communication in *Dictyostelium* cells occurs indirectly through the external medium and is mediated via phosphorelays and second messenger molecules such as cAMP over several minutes. Despite these different architectures and timescales, due to the underlying universal dynamics, both systems can be described by the same model and exhibit qualitatively similar behaviors including noise-induced accommodation spikes. Our work suggests that, like physical systems, collective behavior in biology may be universal and well described using simple mathematical models. Universality has played a fundamental role in furthering our understanding of physical systems, and we suspect it will also play an important role in furthering our knowledge of collective behavior in biology.

## Materials and Methods

### Cell culture, preparation, and genetic manipulation

Axenic *Dictyostelium discoideum* cell lines expressing Epac1camps (AX4 background, gift of Dr. Satoshi Sawai), Epac1camps and mRFPmars (AX4 background), ECFP, and EYFP (both AX3 background, gifts of Dr. Carole Parent) were grown according to standard protocols (Fey *et al*, 2007). Briefly, vegetative cells were grown at 22°C while shaking at 180 rpm in PS medium consisting of 1.0% special peptone (Oxoid), 0.7% yeast extract (Oxoid), 1.5% D-glucose, 0.14% KH_2_PO_4_, 0.012% Na_2_HPO_4_-7H_2_O, 40 ng/ml vitamin B12, 80 ng/ml folic acid, and 1× antibiotic-antimycotic mix (Gibco) supplemented with 5 μg/ml (EYFP/AX3), 10 μg/ml (Epac1camps/AX4), or 20 μg/ml (ECFP/AX3) G418. Vegetative cells were washed and shaken at 1–2 × 10^7^ cells/ml in development buffer (10 mM K/Na_2_ phosphate buffer, 2 mM MgSO_4_, and 200 μM CaCl_2_, pH 6.5) for 4–5 h prior to experiments.

The expression vector pBSRH-mars, which permits constitutive expression of mRFPmars in *Dictyostelium discoideum* under control of the *act15* promoter, was kindly provided by Dr. Robert Cooper. The Epac1camps strain was transformed with pBSRH-mars by electroporation following a standard protocol (Gaudet *et al*, 2007), and a clone was selected based on fluorescence intensity. Fluorescence intensity of mRFPmars appears uniform in the cytosol.

### Microfluidic device fabrication

Microfluidic devices for both single-cell and population experiments were fabricated using standard photolithography techniques to generate silicon masters and standard poly(dimethylsiloxane) (PDMS) replica molding techniques to generate the final devices. For the single-cell experiments, devices with two different feature heights were required to mix the cAMP to provide temporally complex input stimuli (Supplementary Fig S2A) (Stroock *et al*, 2002), and for these silicon masters, we used two-step photolithography (Anderson *et al*, 2000). Briefly, to create the main cell channel, an initial (160 μm thick) layer of SU-8 photoresist was spin coated, exposed to UV light, and developed on a silicon wafer. Subsequently, a second (15 μm thick) layer of photoresist was spun on top of this layer, the first layer features aligned to the mask for this second layer, exposed, and developed. For the population experiments, a 0.8-mm-tall aluminum Y-channel master was machined with 2-mm-wide and 6.5-mm-long input channels and a 3-mm-wide and 19-mm-long cell area.

For both microfluidic devices, the microchannels were formed out of poly(dimethylsiloxane) (PDMS) via replica molding. Specifically, a 10:1 ratio of PDMS pre-polymer to catalyst was poured on top of the master of interest, baked for 50 min at 65°C, and then cut to size and removed from the master. Access holes for tubing were created using a 1.5-mm biopsy punch prior to plasma bonding the slabs to glass coverslips. Devices were then baked for at least 1 h at 65°C to aid in restoring a hydrophobic surface to the PDMS.

### Cell perfusion and cAMP stimulation

For experiments, vegetative cells were harvested at 1.5–3 × 10^6^ cells/ml, washed, and shaken at 1–2 × 10^7^ cells/ml in developmental buffer for 4–5 h before plating inside a microfluidic device. Single-cell microfluidic devices were seeded with 2,000–4,000 cells, and cells were permitted to adhere to the glass for 10 min before a constant flow rate of 4 μl/min was initiated using syringe pumps (Fusion Touch 200, Chemyx). Population devices were seeded with cells to the desired coverage and allowed to rest for 10 min before initiating flow of 10–100 μl/min and imaging 2 mm from the population edge. Macrofluidic dishes were seeded with 0.01 ML (1 ML = 6,600 cells/mm^2^) of cells and allowed to rest for 10 min before initiating flow of 1 ml/min. Cells were maintained at 22°C throughout imaging, and experiments were limited to 130 min to mitigate any potential adverse effects of longer-term perfusion and ensure all cells are in the same developmental stage when a single cAMP receptor is the predominant receptor activating the signaling pathway (Insall *et al*, 1994). For experiments where single-cell cytosolic cAMP traces were taken from a population, 15–20% Epac1camps- and mRFPmars-expressing cells were mixed in the population to facilitate single-cell tracking.

Single cells in microfluidic chips have a higher initial average response than those in macrofluidic dishes (Supplementary Fig S2B and C) as the transition from 0 to the stimulus concentration of cAMP in microfluidic chips is sharp, whereas in macrofluidic dishes, chaotic mixing of the cAMP with buffer leads to a more gradual transition.

### Image acquisition

Cells were observed using an inverted epifluorescence microscope (TE300, Nikon) equipped with a Xenon lamp, automated excitation and emission filter wheels (Ludl), automated stage (Ludl), and oil immersion objectives (20× UPlanSApo NA 0.85, Olympus and 60× Plan Apo NA 1.40, Nikon). For FRET measurements, three fluorescent images were taken at each time point and are described using the following notation: 
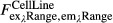
 where *F* is the fluorescence intensity, and the cell line is the line expressing the donor fluorophore only (D, here ECFP), the acceptor fluorophore only (A, here EYFP), or the epac1camps complex (EPAC). The excitation wavelength filter ranges are 436/20 nm for D and 500/20 nm for A, and the emission filter ranges are 470/24 nm for D and 535/30 nm for A (ET series filters, Chroma). A dichroic long-pass filter (T455LP, Chroma) further separated ECFP excitation from emission fluorescence during imaging. Images were captured using a back-illuminated Electron Multiplying CCD (EMCCD) camera (iXon + 897, Andor) with a depth of 16 bits, 256 × 256 resolution, and 1,000× gain. To minimize photodamage, exposure times were limited to 80 ms at 20× magnification and 20 ms at 60× magnification, an ND8 filter was in the emission path, and each field of view was imaged every 15 s. Acquisition was controlled using a custom Java plugin in Micro-Manager (Edelstein *et al*, 2010) that continually centered the cell of interest in single-cell experiments and maintained focus at the plane of interest.

### Data analysis

Image analysis was performed using custom MATLAB (MathWorks) routines. Images were binarized, and for single-cell data, single-cell masks were generated by thresholding each fluorescent cell against its local background. For single cells to be considered the same cell between frames, the current cell location must overlap with the previous cell location and have an area change no greater than 33% to ensure they are remaining in contact with the coverslip. After masking, the 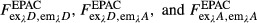 image intensities were averaged across each cell at each time point to reduce noise. To calculate changes in the FRET efficiency, we used the *E f_DA_/γ*-based FRET method (Salonikidis *et al*, 2008). Briefly, *E* is the FRET efficiency, *f_DA_* is the fraction of Epac1camps complexes in their bound states, and *γ* is the relative donor/acceptor extinction. This method improves on the traditional ratiometric FRET method, which uses 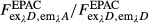 as a readout of FRET efficiency, by correcting for any photobleaching that occurs during long experiments. Furthermore, this method is independent of pH changes unlike the ratiometric method (Salonikidis *et al*, 2008), meaning that any cytosolic pH changes that may occur do not affect our readout of the FRET efficiency. To find *E f_DA_/γ*, we use the formula: 

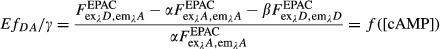
6

where 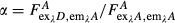, the relative acceptor fluorescence signal, and 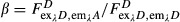, the donor bleedthrough. On our imaging setup, *α* = 0.054 and *β* = 0.906. As Epac1camps is in a low FRET configuration when bound to cAMP and a high FRET configuration when unbound, we use −*E f*_*DA*_/γ as our FRET intensity here. All single-cell and population average FRET intensities are normalized to the baseline levels observed for either the single cell or the population at the beginning of each experiment prior to stimulation (individual cells) or synchronization (population). FRET signal units are defined as the change in −*E f*_*DA*_/*γ* where *E* is the FRET efficiency, *f*_*DA*_ is the fraction of Epac1camps complexes in their bound states, and *γ* is the relative donor/acceptor extinction and 0 is defined as the baseline value of −*E f*_*DA*_/*γ* for the experiment when cells are in cAMP-free buffer.

In our analysis of single cells, the accommodation spike width was defined as the time from the initial rise in cytosolic cAMP after the onset of stimulus to the time where the cytosolic cAMP returned to the baseline cAMP level before rising again for an oscillation or staying at the baseline. The mean oscillation time for single cells was calculated for each concentration by taking the mean of the peak period in Fourier transforms of individual oscillating cell traces, with error calculated through bootstrapping. Entrainment quality for single cells was defined as the mean Pearson correlation coefficient between the intracellular cAMP response to the first pulse of external cAMP and subsequent intracellular cAMP responses to each subsequent pulse of extracellular cAMP. For single-cell analysis of spike width and entrainment quality, cells with clear spikes 0.25 FRET signal units or greater in height were analyzed. When analyzing population average firing rates, spikes were required to be 0.3 FRET signal units in height to be counted as a population spike.

### Simulations

Simulations were done using the Euler–Maruyama method (Kloeden & Platen, 1992) with a time step Δ*t* = 0.005 and started with random initial conditions. For phase diagram calculations, longer equilibration periods were used to eliminate the effect of the initial conditions. Spikes were defined as peaks in activator with values above zero, and rates were calculated by counting these spikes divided by simulation time.

Parameters used for all simulations throughout the entire manuscript (unless stated otherwise) are as follows:

*∈* = 0.1 (ratio between the activator and repressor timescales)

*γ *= 0.5 (repressor degradation rate)

*c*_0_ = 1.2 (steady state repressor value in the absence of external cAMP)

*σ *= 0.15 (noise strength)

*N* = 100 (number of “cells” in population simulations)

*a* = 0.058 (cAMP response magnitude)

*α*_0_ = 800 (basal cAMP leakage rate)

*α_PDE_* = 10^3^(basal PDE leakage rate)

*K*_*d*_ = 10^−5^ (cAMP response threshold)

*S* = 10^6^ (firing cAMP release rate)

## Supporting Information

Source Data for Supplementary Figure S4

Review Process File
