# Supplementary figures and images for: From intracellular signaling to population oscillations: bridging size- and time-scales in collective behavior
Source: Mol Syst Biol. 2015 Jan 23;11(1):779. doi: 10.15252/msb.20145352 (PMC4332153; doi:10.15252/msb.20145352)

Figure S1

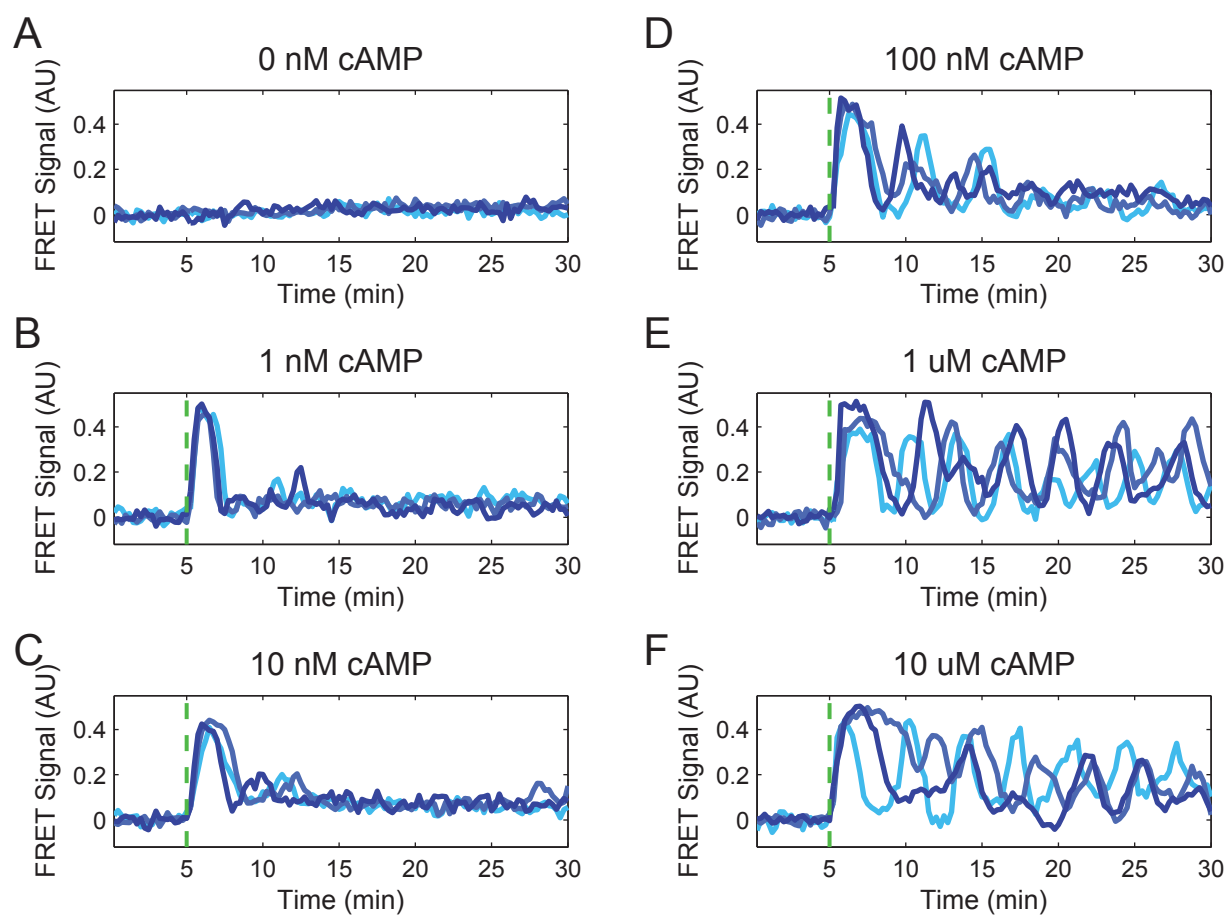

Supplement: Supplementary file 1 [file msb0011-0779-sd1.pdf]

Figure S2

A

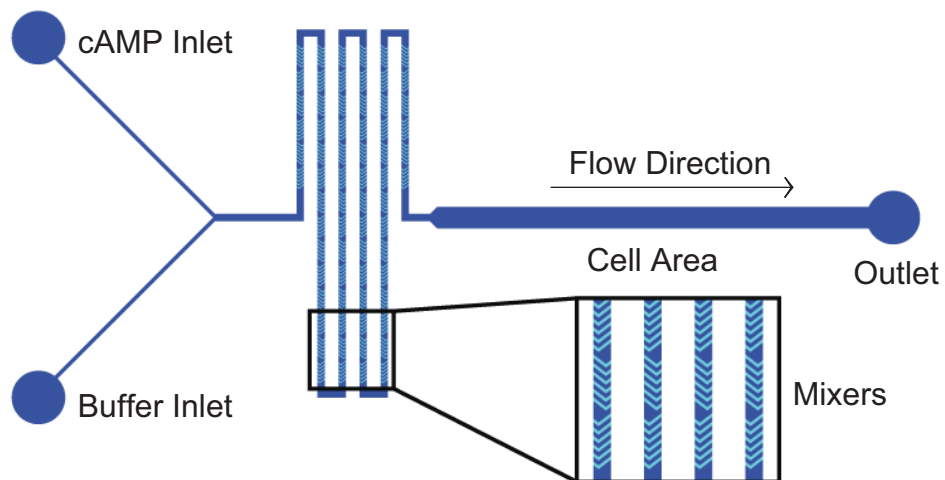

B Microfluidics I/O

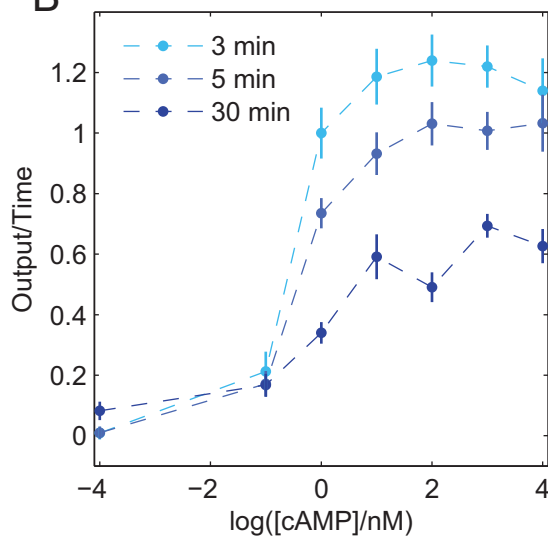

C Macrofluidics I/O

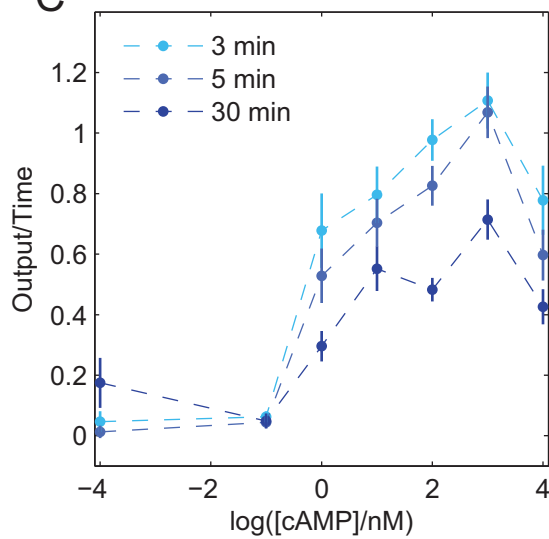

Supplement: Supplementary file 2 [file msb0011-0779-sd2.pdf]

Figure S3

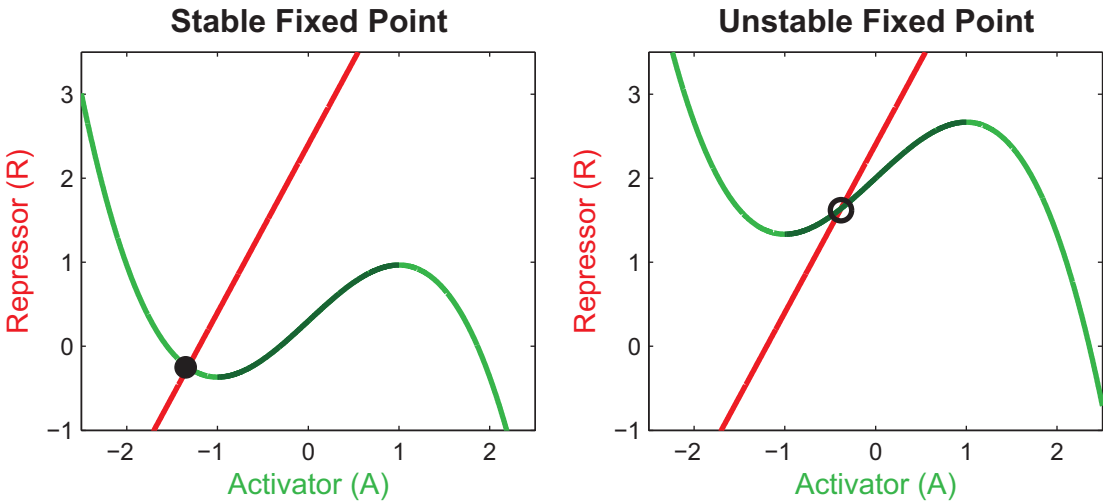

Supplement: Supplementary file 3 [file msb0011-0779-sd3.pdf]

Figure S4

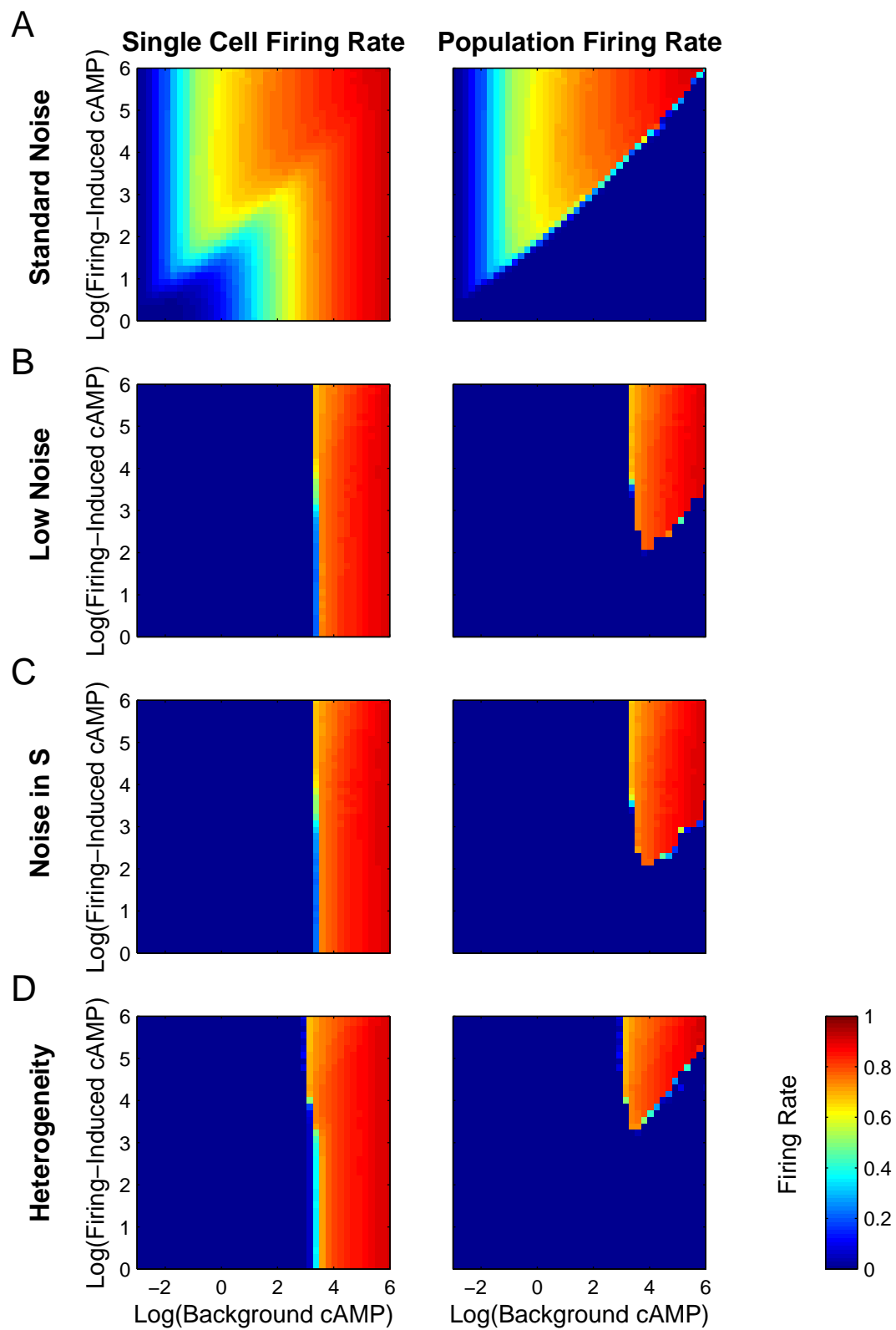

Supplement: Supplementary file 4 [file msb0011-0779-sd4.pdf]
