# Supplementary material for: From intracellular signaling to population oscillations: bridging size- and time-scales in collective behavior
Source: Mol Syst Biol. 2015 Jan 23;11(1):779. doi: 10.15252/msb.20145352 (PMC4332153; doi:10.15252/msb.20145352)
Supplement: Supplementary file 5 [file msb0011-0779-sd5.pdf]

Supplemental Figure S1. Cytosolic cAMP responses to external cAMP stimulus. Cytosolic cAMP responses of three single cells in microfluidic devices to either (A) no externally applied cAMP or an externally applied cAMP stimulus of (B) 1 nM, (C) 10 nM, (D) 100 nM, (E) 1 µM, or (F) 10 µM at 5 min.

Supplemental Figure S2. Microfluidic device characterization. (A) Single-cell microfluidic device with on-chip mixing. Buffer and cAMP are delivered through the inlets, mixed, and delivered to cells in the imaging area. Dark blue channels are 160 µm high and light blue mixing areas are 175 µm high. (B, C) Single-cell input-output ratios of time- and cell-averaged FRET responses in (B) microfluidic devices and (C) macrofluidic dishes for stimuli ranging from 100 pM to 10 µM, quantified for the first 3, 5, and 30 min after stimulation. Output/Time is normalized to the output of cells exposed to a 1 nM cAMP step in a microfluidic chip over the first three minutes of response. Error bars represent SEM.

Supplemental Figure S3. Fixed points and behavior in the FHN model. Phase portrait with activator A nullcline and repressor R nullclines showing different stimulus S values produce different dynamics in the FHN model. At low values of S when the intersection of the nullclines is on the left side of the A nullcline's local minimum, the system has a single stable fixed point (left plot). At higher values of S when the intersection shifts to the right side of the local minimum (shown in dark green), this fixed point becomes unstable (right plot) and oscillations result (see Figure 1H for example).

Supplemental Figure S4. Intracellular stochasticity drives experimentally observed population-level behaviors. Firing rate phase diagrams for single cells in a population and the population as a whole as predicted by our model as a function of background and firing-induced cAMP with (A) standard noise level, $\sigma = 0.15$, equilibration time = 1000, (B) low intracellular noise ($\sigma = 0.01$), equilibration time = 116000, (C) noise in $\frac{d[cAMP]_{ex}}{dt}$ case with $\sigma_A = 0.01$ for the noise in $\frac{dA}{dt}$ and $\sigma_s = 0.15$, where $\frac{d[cAMP]_{ex}}{dt} = \alpha_f + \rho\alpha_0 + \rho S \frac{1}{N} \sum_{i=1}^{N} \Theta(A_i) - D[cAMP]_{ex} + \eta_S$ with η_S an additive Gaussian white noise with a mean of zero and STD $\sigma_s = 0.15$, and (D) heterogeneous population case where K_d values are sampled from a lognormal distribution centered around 10^{-5} with a coefficient of variation of 30%. Model firing rates are normalized to an arbitrarily high frequency (~1/30) to scale maximum values to 1.
