# Supplementary material for: From intracellular signaling to population oscillations: bridging size- and time-scales in collective behavior
Source: Mol Syst Biol. 2015 Jan 23;11(1):779. doi: 10.15252/msb.20145352 (PMC4332153; doi:10.15252/msb.20145352)
Supplement: Supplementary file 9 [file msb0011-0779-sd9.pdf]

From Intracellular Signaling to Population Oscillations: Bridging Size and Time Scales in Collective Behavior

Allyson E. Sgro, David J. Schwab, Javad Noorbakhsh, Troy Mestler, Pankaj Mehta, and Thomas Gregor

Corresponding author: Thomas Gregor, Princeton University

Review timeline:

Submission date:	22 April 2014
Editorial Decision:	21 May 2014
Revision received:	04 October 2014
Editorial Decision:	07 November 2014
Revision received:	07 December 2014
Editorial Decision:	08 December 2014
Revision received:	13 December 2014
Accepted:	16 December 2014

Editor: Maria Polychronidou

Transaction Report:

(Note: With the exception of the correction of typographical or spelling errors that could be a source of ambiguity, letters and reports are not edited. The original formatting of letters and referee reports may not be reflected in this compilation.)

1st Editorial Decision 21 May 2014

Thank you again for submitting your work to Molecular Systems Biology. We have now heard back from the three referees who agreed to evaluate your manuscript. As you will see from the reports below, the referees acknowledge that the presented analysis is potentially interesting. However, they raise a series of concerns, which should be carefully addressed in a revision of the manuscript. The recommendations provided by the referees are very clear in this regard.

If you feel you can satisfactorily deal with the points listed by the referees, you may wish to submit a revised version of your manuscript. Please attach a covering letter giving details of the way in which you have handled each of the points raised by the referees. A revised manuscript will be once again subject to review and you probably understand that we can give you no guarantee at this stage that the eventual outcome will be favorable.

Reviewer #1:

Sgro et al. address the important double problem of (1) how seemingly simple intracellular dynamics give rise to complex outputs at population level, and (2) how to model such phenomena when we have limited knowledge of their molecular details. In this study, the authors focused on the starvation response in Dictyostelium and analysed the progression from transient pulses of intracellular cAMP (in response to low extracellular cAMP) to sustained oscillations (in response to higher levels of extracellular cAMP). This is a well-known and established system, but many of its details are still unclear.

Using time-lapse microscopy of FRET reporter strains and mathematical modelling, the authors obtained new interesting insights into the dynamics of this system, and found it is possible to explain and predict a number of observations - at both single cell and population levels - by using a general phenomenological model. Specifically, the authors borrowed the classic Fitzhugh-Nagumo (FHN) model from systems neuroscience, and showed this model can explain adaptation spikes in single cells at sub-threshold levels of external cAMP, sustained oscillations above threshold and synchronised collective waves of cAMP release. This is not the typical and static sensing threshold: different dynamics of input stimulation cause very different dynamic outputs.

All these observations are consistent with assuming Dictyostelium's signalling architecture can be projected into an effective lower dimension circuit that is excitable - the FHN circuit. The authors make a compelling case for their strategy: using so-called "universal" models to characterise biological systems across multiple scales of abstraction, provide quantitative insights, and make testable predictions.

This study will therefore appeal to not only cell biologists interested in signalling dynamics in Dictyostelium, but also to biologists and physical scientists interested in quantitative approaches to signalling and regulation in diverse organisms. I am not an expert in Dictyostelium signalling, so I defer to other reviewers on the cell biology aspects of the paper. I have the following major and minor points which I would like to see revised.

Major points:

1. It would improve the paper to strengthen the findings concerning the rate responsiveness of the system. In Fig. 3B, in the experimental traces panel, there appears to be a pulse after 30 minutes. The amplitude of this pulse is comparable to that of the initial accommodation spike of Fig. 3A. What is the origin of this pulse? It seems it might be related to the start of application of the ramp, in which case it is slightly at odds with the main conclusion of the figure. Please discuss why this pulse is present. It might also be useful to replicate this experiment with a rate that is, e.g., twice the rate of Fig. 3B. The system should then still be largely insensitive to the rate of change, but reach the super-oscillation threshold at an earlier time.

2. It is not clear how closely the experimental output matches an excitable dynamical system. Please comment on why the 3 representative traces of the 1 minute step in Fig. 4A appear significantly more synchronised during the first pulse than the 3 traces of the 5 minute ramp. If the system is excitable this should not be the case: following initial application of the stimulus, both systems should be insensitive for the duration of the refractory period, unless this is very short.

3. The description of how noise is driving population behaviour is unclear. In Fig. 7C and page 16 of the main text, standard deviation is used as a metric of stochastic variation. It is stated that the "standard deviations are normalized to mean standard deviation at 10nM external cAMP". It would be helpful to have this normalisation clarified. How would the results change if a different metric, such as the coefficient of variation (or the Fano factor) is used?

4. The authors often refer to the "universal" properties of the model, and therefore the choice of parameters is of secondary importance to the qualitative predictions. Nevertheless, the model simulations are parameterized. Please discuss, either in the text or in a supplement, the choice of parameters.

Minor points:

1. In the abstract, the authors state the model explains effects at "multiple spatial and temporal scales", but no explicit spatial modelling has been presented. Please modify this sentence or support it with more data.

2. Please avoid the use of adjectives such as "remarkable", "striking", etc.

3. It is well established that the FHN model generates sustained single neuron spikes (as you state), as well as sustained oscillations in a neural network for certain regimes of inputs and interactions. It should probably be acknowledged when discussing the population level model and oscillations. This

© European Molecular Biology Organization

in no way undermines the importance and novelty of the approach and results.

4. The 4th line in the 2nd paragraph of page 7 was likely meant to refer to Fig. 1D, not 1C.

5. The variable S is not defined when the population model is presented in page 12.

6. In page 13, is the firing induced cAMP meant to read rho*S/J, rather than rho*D/J?

7. It is unclear what is meant by "cells lacking extracellular cAMP" in page 18: is it cells lacking intracellular cAMP or the ability to sense extracellular cAMP?

8. In page 26, specify units of parameters. If normalised to be non-dimensional, please state the normalisation operation.

9. In page 33, the caption for panel 1C appears to have the blue hues wrong (dark blue for 2nM)

10. In page 34, state which trace corresponds to which excitability.

11. Please provide details of the calculation (with the relevant equations) of entrainment quality, peak width, mean oscillation times, etc. in materials and methods or in a supplement. For example, is mean oscillation time the mean of the peak period in Fourier transforms of individual traces, or the peak period in the Fourier transform of the mean?

12. In Fig. 6D, it would be interesting to see the experimental single cell traces, either here, or perhaps in a supplementary figure (so as to not overload this figure).

13. Is it possible to use a different term rather than adiabatic in the text? Adiabatic processes can be achieved in different ways in different areas of physics, and its usage here does not help make the text clear for a general audience.

14. In the printed version it is sometimes difficult to distinguish between hues of the same colour (e.g. multiple shades of blue in Figure 1A). Would it be possible to change this?

15. Figure 7A has no intensity bar.

Reviewer #2:

In their manuscript "From Intracellular Signaling to Population Oscillations: Bridging Scales in Collective Behavior", Sgro and coauthors use a generic FitzHugh-Nagumo (FHN) model of an excitable system for description of single-cell and collective cAMP oscillations in Dictyostelium discoideum (Dicty). The paper is well written, and the line of thoughts is presented in a clear manner. However, we cannot recommend the publication of the manuscript without a major revision. Our main criticism is related to the authors' claim that their general approach to the analysis of a biological system such as Dicty in the framework of a generic model is a novel idea. In fact, this type of modeling has been extensively used in multiple areas of biology (generic low-dimensional models and networks in neuroscience, generic excitable media models for cardiac dynamics, etc). In the Dicty field, generic models of diffusively coupled simple excitable elements were successfully applied to studies of cAMP spiral waves and complex spatio-temporal patterns of amoeba aggregation. Furthermore, even FHN-based systems were applied to modeling of Dicty before, albeit indeed in a somewhat different form. However, the relevant papers (e.g. Vasiev, Maree) were only cited at the very end, in the Discussion. All these studies should be mentioned right up-front, in the Introduction. Otherwise, all the way through the results, the reader is left with the false impression that this is the first use of FHN (or even the idea of modeling Dicty as an excitable system) in the context of Dicty. In our view, the contribution of this work is more about taking an idea that was already out there (Dicty as excitable/oscillatory FHN-type systems) and building a more accurate model of that sort on the basis of additional experimental data (single-cell dynamics, external time-dependent cAMP excitation). If this impression is wrong, the authors need to explain clearly why their approach is not comparable to previous attempts to model Dicty behavior as generic excitable/oscillatory systems.

© European Molecular Biology Organization

There are several other related points that also highlight shortcomings of this m/s that need to be addressed:

1) A problem arises when the authors talk about the "striking agreement" between the supposedly simple model and the experimental observations. On the one hand, the authors emphasize repeatedly that they exploit universality and that their results do not depend on the actual choice of the model. One main point they are trying to make is that Dicty cells are not threshold sensors but in contrast, their spiking is due to an internal state reaching a threshold, and they provide experimental evidence for this by showing that the same level of cAMP can cause a spike or not depending on history (Figure 3A). However, the authors themselves mention on page 6 that this property is "specific to the FHN model". In this context it sounds contradictory that the "qualitative predictions do not depend strongly on the choice of parameters and the form of the nonlinearity f(A)" (page 7). A discussion about how universal this property actually is for excitable systems is needed here in order to make the universality claim. The same is true for the parameter choices: the FHN model is very versatile and can be tuned into a number of qualitatively different regimes and also can be varied quantitatively in terms of spike duration, refractory time, time scale separation, etc. So it needs to be discussed how the specific choice of parameters and the choice of the input function I(x) impact the universality claim.

2) The evidence in Figure 3 is not convincing to us. In their discussion of Figure 1B on page 8, the authors acknowledge that, in reality, there are long-term adaptation process altering the response of the cells. The time scale on which this is happening is on the order of tens of minutes as shown in Figure 1B. However, the time scale of the slow rise of cAMP shown in Fig. 3A is equally long. Couldn't the same mechanism be at work here? Then, the observed behavior would not be a result of the quasi-static change of the system state with the fixed point (as discussed on page 9) but of something that is not captured by the model. More evidence is needed here. Also, the numerical time course in Fig. 3B does not show any oscillations until the very end when external cAMP is close to the final value of 300nM. This is surprising since according to Fig. 1, the bifurcation to the oscillatory dynamics occurs at much lower value of cAMP <10nM. How can these two pieces of numerical evidence be reconciled? Or have two different sets of parameters been used there?

3) The idea of noise-induced synchronization of accommodation spikes across the population is certainly interesting. It very much resembles the phenomenon of coherence resonance on a population level (the revised manuscript should certainly connect to the coherence resonance literature). The question that arises here and should be discussed is: how important is this mechanism for the actual spatial organization of activity at the colony level? It is plausible that, once pattern formation comes into play and cAMP waves move through the colony to deterministically cause "spiking" of individual cells, the oscillatory behavior will be the result of general excitable-medium type mechanisms (e.g., spiral waves) and not due to intracellular noise The authors should explain in what situation they think their colony synchronization mechanism could be at work. On page 18, they compare their results to the studies investigating spiral wave behavior of the population. But this situation could be fundamentally different than a whole colony oscillating in synchrony (due to the above reasons).

5) the phrase "Bridging Scales in Collective Behavior" in the title does not adequately describes the scope of the manuscript. In fact, only one scale of collective behavior is actually investigated, namely the global coupling through a medium (that has no associated length scale). In our view, bridging the scales in collective behavior would require to use the proposed model in the spatio-temporal context in order show that it actually produces testable predictions for the spatial organization at the colony level that differ from earlier phenomenological models that already predict spiral wave behavior and amoeba aggregation patterns. Generally, that should be expected of the paper suggesting a new model of Dicty dynamics, given the rich history of successful spatio-temporal models in this field.

To conclude, in our opinion the paper needs a major revision.

Reviewer #3:

In their manuscript, Sgro and colleagues present a new model, nicely validated by experiments, of the single-cell and collective behavior of the social amoeba Dictyostelium discoideum. The choice of this new model is motivated by the observation that the behavior of single Dictyostelium cells in

response to cAMP bifurcates from single intracellular cAMP pulse at low concentration, to intracellular cAMP oscillations at high extracellular cAMP concentrations. Making a parallel to the excitable behavior of neurons, the authors use a 2D FitzHugh Nagumo (FHN) model to characterize this behavior. In a nice series of experiments, the authors validate this model choice and then use it to build a model of the collective behavior of a population of Dictyostelium cells. Using this model of collective behavior and additional experiments, they present evidence for the role of noise - the stochasticity in the pulse response to extracellular cAMP - in the entrainment of a collection of Dictyostelium cells toward a synchronously oscillating population.

With their overall very well-done study, the authors present a new conceptual understanding of Dictyostelium behavior. While the short time-scale response to cAMP was previously ascribed to an incoherent feedforward (Takeda et al., 2012), here the analysis of the slower time-scale behavior reveals the importance of feedback (negative and positive) in driving the experimentally observed Dictyostelium cellular behaviors. The approach taken, with a very simple model based on the key emergent qualities of the cellular behaviors, has been used do explain other behaviors of Dictyostelium, but is conceptually new for the investigation of the oscillatory behavior of the response to cAMP and could also potentially apply more broadly to understand how single-cell responses might drive collective behaviors. In general, the results are conclusions are validated by well-designed experiments, although to further strengthen the manuscript a few key points should be addressed:

1) It seems to me that there are three behaviors (instead of just two) for the response of single Dictyostelium cells to extracellular cAMP concentrations: a single pulse at low concentration, stochastic pulses at intermediate concentrations, then oscillations at high concentrations (Figure 2 of Gregor et al. 2010) - is the behavior at intermediate concentrations also well accommodated by the FHN model?

2) Using the results presented in Figure 1D the authors state: "Experimentally, we find that the upstream circuit senses fold-changes in cAMP...", however the experiment is not a strong demonstration of fold-change detection because the two-fold change is done at overall lower cAMP concentration than the 10-fold change. Fold-change sensing would require showing that a two-fold change resulting in the same final 10 nM concentration (so 5 nM to 10 nM) elicits the same response as the change from 1 nM to 2 nM cAMP. Another suggestion would be for the authors to do a three-step experiment, comparing the response to: 1 nM to 2 nM to 20 nM (two-fold, then 10-fold) and 1 nM to 10 nM to 20 nM (10-fold then two-fold, but arriving at the same final concentration of cAMP). Finally, it is not clear from the text whether fold-change sensing is an important emergent property of a FHN system.

3) Figure 3B - A bit more should be said in the mis-match between model and experiments in this particular scenario. Is the excitability of the cells even greater than that of the two instances of the model? What else might explains the observed pulsing behaviors of the single cells during cAMP ramping, that is not observed in the model? Also all three cells were observed to spike twice at around 30 min (the model does not) - what concentration is reached at that time point? What other experimental variable might explain these spikes if these events precede the initiation of the ramping up of cAMP?

4) Figure 6A - is there an experimental justification for (a priori) choosing the particular position I (and therefore the relative positions II, III and IV)? Perhaps it would be more natural to describe these results as using the experimental data (in C and D), to position Dictyostelium cells within that region of the phase diagram of the system (i.e. using data to learn about a plausible parametrization)?

5) P. 15 - to test whether cell-to-cell heterogeneity in the threshold of extracellular cAMP needed to induce a cytosolic spike, the authors use a mixture of only two different model parametrizations. Because heterogeneity in this threshold could be due to variability in the level of a key sensing protein in the system, a better representation of the heterogeneity might be to sample Kd from a lognormal distribution of value centered around the nominal value (10e-5). An appropriate standard deviation would be 25-30% (typical CVs for protein level heterogeneity are 15-50%). Using this representation of heterogeneity is the same conclusion reached?

Minor concerns:

1) P. 4 - The final sentence of the introduction ends with "describe both single cell and multicellular dynamics in collective biological systems." Because the phrase "collective biological systems" is relatively vague, it might actually help to include specific examples.

2) P.7 - It might help the general readership to more directly define the expression "accommodation spikes" as it is an important concept in the context of this model.

3) P.8 - the text states "Notice that the model reproduces the initial accommodation spikes for all values of externally applied cAMP followed by oscillations for the 10 uM stimulus." Although the traces presented in Figure 1 (G, H) are only for two values of cAMP (1nM and 1 uM); adding more traces in a supplementary figure would strengthen that point.

4) P. 8 - "At longer time scales (> 10 min), genetic regulation becomes a factor in our experiments, causing oscillation periods to change and possibly down-regulating noisy firing." Is what is meant here that in a single-cell, new gene expression at that longer time scale would change its behavior over time? (In contrast to cell-to-cell variability in the expression of certain genes causing cell-to-cell variability in long time-scale behaviors?); rephrasing may help clarify.

5) Figure 1E-F - In the figure legend, the reference to the green lines is swapped I believe (dark green should be the baseline condition, not the light green; although in that case the line colors are reversed with those used in Figure 3C-D where baseline is light green).

6) P.11, 12 - for the more general readership, it would be helpful to qualify a bit further the cAMP release parameters in the model. If I understand correctly 0 describes a low-level baseline, constant release of cAMP, while S describes the additional release of a larger concentration of cAMP that only accompanies a spike?

7) Figures 4B-C, 5A-B, 6A,C and 7A; it might be even more informative to use single-color gradations (or potentially two-color gradations, see comment below for Figure 6C) rather than a cold-warm color scale in each of these heatmaps.

8) P.15 - the authors state "when one or a few cells stochastically spike and drive the rest of population into synchrony." Is there a requirement for a few cells to spike nearly simultaneously for entrainment to begin? If so, it may be more accurate to state: "when one of a few cells stochastically spike at around the same time, they can drive the rest of the population into synchrony."

9) P.16 - Figure E3D seems mislabeled on the figure itself (as panel "S"?)

10) Figure 2B - The legend notes that the colored (blue) dots are from the data plotted in 2A; the two additional dots are from additional data collected at other cAMP concentrations? The error bars are said to represent SEM, for an n = ?.

11) Figure 5, legend - The sentence beginning with "Low flow rates where the effect..." is awkward please simplify (perhaps "Low flow rates are note plotted because in those conditions the effect of extracellular PDE is non-negligible.")

12) Figure 6A - the labels and dots (I, II, III, IV) are very hard to see on the population firing rate heatmap.

13) Figure 6D - it would be helpful to add on the plots of the data the actual concentrations of added cAMP

1st Revision - authors' response 04 October 2014

(see next page)

2nd Editorial Decision 07 November 2014

Thank you again for submitting your work to Molecular Systems Biology. We have now heard back from the three referees who were asked to evaluate your manuscript. As you will see below, while the reviewers think that their main concerns have been satisfactorily addressed, they list a number of relatively minor concerns, which we would ask you to address in a revision of the manuscript.

Some the remaining issues are related to modifications in the text and some of the figures. Moreover, reviewers #1 and #2 refer to the need to discuss alternative explanations that could exist for some of the behaviors that were observed experimentally. Please note that we have also circulated the reports to all reviewers as part of our 'pre-decision cross-commenting' policy. During this process, reviewer #3, mentioned that s/he agrees with reviewers #1 and #2 that discussing alternative explanations is important "especially for 1) spike amplitude and activity-induced gene regulation and 2) spike wave form and cell shape, where the explanation provided is possible but not validated in this paper". Reviewer #3 also mentioned that, regarding the ramping rates (Figure 3), "it would be helpful to state more clearly whether the 'system' with limitations is the experimental set-up (technical) or the experimental model (biological)".

Reviewer #1:

Although I appreciate the attempts to address our questions by the authors, I am still confused by some of the responses, and would appreciate more details before agreeing to publication.

My main concerns lie in the response to two of our major concerns about the paper.

1. The rate responsiveness of the system

The authors claim that they can not do faster or slower ramps due to the limitations of the system. For fast rates they claim the system are limited to 3nM step sizes. This seems a little confusing, as they use 1nM step sizes in Fig 3A (in fact smaller than 1nM for the ramp). I am assuming that the 3nM step limit is because of the final ramp amplitude being up to 300nM in Fig 3B. Could the authors go up to a different maximum ramp amplitude and then do faster ramps to examine rate responsiveness?

For slower rates, the authors state that 'Slower ramps move beyond the time window starving Dictyostelium cells use one set of cAMP receptors to respond to stimuli and into a different developmental regime.' This raises the question of how the authors have checked that the Dictyostelium cells are using one set of cAMP receptors to respond to stimuli for their chosen ramp time? Also, in the text in another context, explaining why the model doesn't match the data completely (Page 9 paragraph 1), the authors write ' We suspect that this is due to genetic regulation becoming a factor in our experiments at these longer (>10 min) timescales' . Does this suggest that genetic regulation is a factor in the 1 hour long ramps chosen? It would be useful to have a discussion of these limitations in the text, which is currently missing. I am worried how general the results can be, if it is really possible to only do one ramp.

We note that reviewer 2 also had concerns about Figure 3. I am confused by the footnote added in response to their question, which I think general readers might also find confusing.

""While the timescales involved in these ramp experiments are long, cells remain quiescent during the majority of the experiment. Thus the activity-dependent gene regulation that causes our model predictions to diverge from experimentally observed behaviors (e.g. oscillation dampening) is not an issue in these experiments."

I am confused by what the authors mean by the cells being quiescent during the majority of the experiment. Does this mean that there is no protein translation ? How can we determine when the cells are quiescent or not in the experiment, and how does this fit with their model?

2. How well the output matches an excitable dynamical system.

We were concerned about the change of waveform shape between a 1 minute step (Fig 4A top) and

a 5 minute step (Fig4A bottom). For an excitable system, once the system is kicked on, the pulse shape should be stereotyped.
The authors respond by stating: 'We suspect this effect is due to cells that do not remain flat on the surface of the coverslip when becoming rounded upon stimulation with cAMP (Alcantara and Monk, 1974). While we discard cells that undergo a dramatic reduction in size, indicating that they are now in poor contact with the coverslip, we require that this change in size be quite large and thus we do have data from cells that did not maintain perfect contact with the coverslip and thus appear to give less well synchronized responses.'

This response raises questions about the experimental techniques. I would have thought it would be possible to go back through the data and examine whether they can systematically find a difference between the focus of cells from Fig 4A top compared to Fig4A bottom? It worries me that the focus difference can have this large an effect, and the note that the authors add on Page 26 does not explain that the traces in the data can be from out of focus images. How is peak width affected by the focus changes? Could it affect the results in Fig 3B peak width?

Reviewer #2:

Many thanks to Sgro et al. for revising the manuscript and their detailed answers to all our and the other two reviewer's comments/questions. We think the manuscript has significantly improved in terms of the motivation of the strategy, connection to existing literature, explanation of the method and discussion off the possible scenarios, in which their results could play a role. We would like to recommend it for publication, under one condition that concerns one of our previous questions:

We were concerned that the long-term adaptation process responsible for the decline in amplitude during oscillations could also play a role in the results of ramp experiments shown in figure 3. The authors explained to us (and do so on page 9 and a footnote on page 10 of the revised manuscript) that they think the dampening of oscillations and down-regulation of noise firing are caused by an activity-dependent adaption process that therefore plays no role for the ramp during which cells are silent. However, this mechanism of adaptation is hypothetical and only one of several possibilities. What if the adaptation is also, or predominantly cAMP-dependent instead of being only activity-dependent? For example, the variation in amplitudes in figure 7D (pulsed cAMP-stimulation with different periods) can only partly be explained by the refractoriness and transient behavior of a simple excitable system, and there might well be other kinds of adaptation in action here.

In addition, there seems to be a disparity in the noisy firing behavior between the model and experiment, if one compares the single cell traces in figure 7B with the simulated single cells in figure 6B at higher cAMP levels (high frequency noise for the former and unsynchronized, yet distinct, pulses for the latter). Nevertheless, we acknowledge the authors' explanation for this effect (top of page 18 of the manuscript) and would like to add that, probably, measurement noise does not allow for a definitive assessment of single cell behavior in this case.

In our view, the authors' choice not to model the adaptation behavior causing dampening of oscillations (also expressed in the answer to point 1 of Reviewer #3) and the disparity in single cell behavior in unsynchronized populations lead to an uncertainty in the mechanism of single-cell dynamics and population synchronization behavior. Therefore, we would like to ask the authors to state in their discussion/conclusion more clearly that there is room for other possible explanations of experimentally observed population-level behaviors. If the authors better described the limitations of their model and resulting caveats in their conclusions, this will, at the same time, strengthen the results for which there is conclusive evidence.

We leave it up to the editor to decide whether our request has been fulfilled in the next revision of the manuscript.

Reviewer #3:

I am pleased to see that with their manuscript revision, Sgro and colleagues have addressed most of the concerns raised in the initial reviews. New data and several clarifications throughout the revised

manuscript have further strengthened the paper, helping, in particular, to explain pieces of data that had previously seemed to undermine some of the interpretations in the original draft. Overall, the manuscript is clear, well written and the model that is presented is well supported by several validation experiments.

A few suggestions for additional minor changes:

1. Regarding Reviewer 1's second point - the authors could add a brief statement to the result section addressing the issue that although an excitable system is expected to produce very similar spikes in all cells, changes in cell shape may be what causes different cells to have differently shaped spikes upon stimulation with cAMP. Although they now address how they deal with cells that reduce contact with the coverslip in the methods, readers it's a sufficiently important point to be brought up in the description of the results.

2. P.36, caption for figure 1. The caption only describes 2 of the 3 curves in the panel.

3. P.25, bottom paragraph. "higher initial average response than those in microfluidic dishes..." - should this be "macrofluidic"?

4. In multiple figures, there are green dashed vertical lines indicating the start of treatment, but although they are visible on my screen, they did not print (may be too thin?)

5. It would be helpful to indicate in each figure caption whether the experiment was performed in the micro- or macro-fluidic setup (which would explain, for example, the three orders of magnitude difference in flow rates in figure 5 vs. figure 7).

2nd Revision - authors' response 07 December 2014

Reviewer #1:

Although I appreciate the attempts to address our questions by the authors, I am still confused by some of the responses, and would appreciate more details before agreeing to publication.

My main concerns lie in the response to two of our major concerns about the paper.

1. The rate responsiveness of the system

The authors claim that they can not do faster or slower ramps due to the limitations of the system. For fast rates they claim the system are limited to 3nM step sizes. This seems a little confusing, as they use 1nM step sizes in Fig 3A (in fact smaller than 1nM for the ramp). I am assuming that the 3nM step limit is because of the final ramp amplitude being up to 300nM in Fig 3B. Could the authors go up to a different maximum ramp amplitude and then do faster ramps to examine rate responsiveness?

For slower rates, the authors state that 'Slower ramps move beyond the time window starving Dictyostelium cells use one set of cAMP receptors to respond to stimuli and into a different developmental regime.' This raises the question of how the authors have checked that the Dictyostelium cells are using one set of cAMP receptors to respond to stimuli for their chosen ramp time? Also, in the text in another context, explaining why the model doesn't match the data completely (Page 9 paragraph 1), the authors write ' We suspect that this is due to genetic regulation becoming a factor in our experiments at these longer (>10 min) timescales' . Does this suggest that genetic regulation is a factor in the 1 hour long ramps chosen? It would be useful to have a discussion of these limitations in the text, which is currently missing. I am worried how general the results can be, if it is really possible to only do one ramp.

We note that reviewer 2 also had concerns about Figure 3. I am confused by the footnote added in response to their question, which I think general readers might also find confusing.

""While the timescales involved in these ramp experiments are long, cells remain quiescent during

the majority of the experiment. Thus the activity-dependent gene regulation that causes our model predictions to diverge from experimentally observed behaviors (e.g. oscillation dampening) is not an issue in these experiments."

I am confused by what the authors mean by the cells being quiescent during the majority of the experiment. Does this mean that there is no protein translation ? How can we determine when the cells are quiescent or not in the experiment, and how does this fit with their model?

The use of syringe pumps does not experimentally limit us to a particular step size, but to step sizes that are a percentage of the final step height, and thus the reviewer is correct that the 3 nM step (1% of 300 nM) is due to the final step height of 300 nM. We find that faster ramps do not produce an adiabatic-type response while slower ramps take so long that cells may move out of the developmental time window we are examining. Ramps to different heights over the same time range either are not sufficiently high to cross the oscillation threshold or are so high that the initial steps given with the syringe pump drive cells over the oscillation threshold shortly after the beginning of the ramp period. We realize these experimental limitations do limit the generalizability of the result shown in Figure 3B and have added a statement to the text on page 11 paragraph 2 emphasizing that we are limited in our ability to probe the full range of possible behaviors in this experiment. The text now reads “While we are limited both experimentally through the use of syringe pumps and by the time window of the developmental phase we are examining in our ability to probe different ramping speeds and heights that may affect the generalizability of this result, we again find that single cells can be sensitive to the rate of change of stimulus as our model predicts (Figure 3B).”

The use of different receptors during different times in development in *Dictyostelium* is documented in the literature, with the primary cAMP receptor being cAR1 during early development and other cAMP receptors, primarily cAR3, becoming more active during aggregation and later developmental stages. Both cAR1 and cAR3 activate common cAMP signaling pathway components leading to cAMP production, so we limit the time of our experiments to avoid the aggregation stage of development. We have added a statement to this effect to our methods section, which on page 26 paragraph 3 through page 27 paragraph 1 now reads “Cells were maintained at 22 °C throughout imaging and experiments were limited to 130 minutes to mitigate any potential adverse effects of longer-term perfusion and ensure all cells are in the same developmental stage when a single cAMP receptor is the predominant receptor activating the signaling pathway (Insall et al, 1994).”

Cells that do not respond with changes in their cytosolic cAMP during an experiment, such as during these slow ramps, are in a state of inactivity or quiescence. This term is not meant to comment on protein translation or other phenomena, merely whether or not cells are responding to external cAMP stimuli with changes in their own cytosolic cAMP levels. We have further modified the text to both comment in more detail on possible genetic regulation when we expose cells to cAMP, and clarified our footnote regarding activity-dependent gene regulation. Specifically, page 9 paragraph 1 now reads "We suspect that this is due to genetic regulation becoming a factor in our experiments at these longer (> 10 min) timescales as *Dictyostelium* is known to regulate gene expression based on cAMP exposure and stimulus shape (Mann & Firtel, 1989). While adaptation processes are clearly at work over longer timescales, in this work we focus only on the shorter time dynamics in an effort to understand the dynamical mechanisms underlying the signal relay response for a given adaptation state." Our footnote now reads “While the timescales involved in these ramp experiments are long, cells remain quiescent and do not respond to cAMP stimulus with production of their own cytosolic cAMP during the majority of the experiment. Thus we speculate that the activity-dependent gene regulation that causes our model predictions to diverge from experimentally observed behaviors during long, constant exposure to cAMP (e.g. oscillation dampening) is not an issue in these experiments. Our suggestion of activity-dependent adaptation is only one possible explanation of the slow ramp behavior, and we leave a detailed investigation to future work.”

2. How well the output matches an excitable dynamical system.

We were concerned about the change of waveform shape between a 1 minute step (Fig 4A top) and a 5 minute step (Fig4A bottom). For an excitable system, once the system is kicked on, the pulse shape should be stereotyped.
The authors respond by stating: 'We suspect this effect is due to cells that do not remain flat on the

© European Molecular Biology Organization

surface of the coverslip when becoming rounded upon stimulation with cAMP (Alcantara and Monk, 1974). While we discard cells that undergo a dramatic reduction in size, indicating that they are now in poor contact with the coverslip, we require that this change in size be quite large and thus we do have data from cells that did not maintain perfect contact with the coverslip and thus appear to give less well synchronized responses.'

This response raises questions about the experimental techniques. I would have thought it would be possible to go back through the data and examine whether they can systematically find a difference between the focus of cells from Fig 4A top compared to Fig4A bottom? It worries me that the focus difference can have this large an effect, and the note that the authors add on Page 26 does not explain that the traces in the data can be from out of focus images. How is peak width affected by the focus changes? Could it affect the results in Fig 3B peak width?

There is a difference between cells being in focus and cells being in contact with the coverslip, such that their intracellular volume is predominately in a single focal plane. All experiments are conducted with regular correction for focus and when cells are not kept in focus, they are not used for analysis. However, as described in our previous response, *Dictyostelium* cells are known to change the amount of contact they have with a surface in response to cAMP stimulation. In order to ensure we are able to correctly assess peak width and distinguish effects cell shape change may have on our ability to distinguish a peak from the baseline, we limit our quantitative analysis of accommodation spikes in Figure 2B and entrainment in Figure 4 to clear spikes 2.5 FRET or units or greater in height. This has been added this to the methods section on page 29, paragraph 2. We have also added a statement to the text concerning this issue. Specifically, we now state on page 23 paragraph 1, "Finally, we note that the FHN model does not reproduce the experimentally-observed spike shape and that the experimentally-observed spike shape does not always match the average, stereotyped response, likely due to cells that do not remain flat on the surface of the coverslip when becoming rounded upon stimulation with cAMP (Alcantara & Monk, 1974)."

Reviewer #2:

Many thanks to Sgro et al. for revising the manuscript and their detailed answers to all our and the other two reviewer's comments/questions. We think the manuscript has significantly improved in terms of the motivation of the strategy, connection to existing literature, explanation of the method and discussion off the possible scenarios, in which their results could play a role. We would like to recommend it for publication, under one condition that concerns one of our previous questions:

We were concerned that the long-term adaptation process responsible for the decline in amplitude during oscillations could also play a role in the results of ramp experiments shown in figure 3. The authors explained to us (and do so on page 9 and a footnote on page 10 of the revised manuscript) that they think the dampening of oscillations and down-regulation of noise firing are caused by an activity-dependent adaption process that therefore plays no role for the ramp during which cells are silent. However, this mechanism of adaptation is hypothetical and only one of several possibilities. What if the adaptation is also, or predominantly cAMP-dependent instead of being only activity-dependent? For example, the variation in amplitudes in figure 7D (pulsed cAMP-stimulation with different periods) can only partly be explained by the refractoriness and transient behavior of a simple excitable system, and there might well be other kinds of adaptation in action here.

In addition, there seems to be a disparity in the noisy firing behavior between the model and experiment, if one compares the single cell traces in figure 7B with the simulated single cells in figure 6B at higher cAMP levels (high frequency noise for the former and unsynchronized, yet distinct, pulses for the latter). Nevertheless, we acknowledge the authors' explanation for this effect (top of page 18 of the manuscript) and would like to add that, probably, measurement noise does not allow for a definitive assessment of single cell behavior in this case.

In our view, the authors' choice not to model the adaptation behavior causing dampening of oscillations (also expressed in the answer to point 1 of Reviewer #3) and the disparity in single cell behavior in unsynchronized populations lead to an uncertainty in the mechanism of single-cell dynamics and population synchronization behavior. Therefore, we would like to ask the authors to state in their discussion/conclusion more clearly that there is room for other possible explanations of experimentally observed population-level behaviors. If the authors better described the limitations of their model and resulting caveats in their conclusions, this will, at the same time,

strengthen the results for which there is conclusive evidence.

We leave it up to the editor to decide whether our request has been fulfilled in the next revision of the manuscript.

We have added a number of statements to the text to clarify our comments on adaptation as stated in our comments to Reviewer 1. Additionally, we have added a new paragraph to the discussion section to clarify the limitations of our model and results due to the lack of adaptation and emphasize which results the model strongly supports. Specifically, page 22 paragraph 2 through page 23 paragraph 1 now reads “We emphasize that we have neglected several phenomena in crafting our simple model and that these simplifications leave open the possibility that there may be alternative models that also explain our single-cell and population level data. It is likely that due to changes in gene expression during development, many molecular components many vary on the timescale of hours (Mann & Firtel, 1989). In our model, this could manifest in a number of ways, including changes in the value of parameters with time as well as minor changes in the shape of the corresponding nullclines. We also have ignored the dynamics of adaptation mechanisms and modules that lie upstream of our excitable circuit. This is one possible reason why our model does not reproduce the damped oscillations observed in response to prolonged stimuli of cAMP. However, it is worth emphasizing that there may be other equally plausible explanations for these experimentally observed behaviors. Our model also does not distinguish between the activator variable, A, and the internal levels of cAMP produced when the activator is spiking. This distinction may be important for understanding certain phenomena such as adaptation. Finally, we note that the FHN model does not reproduce the experimentally-observed spike shape and that the experimentally-observed spike shape does not always match the average, stereotyped response, likely due to cells that do not remain flat on the surface of the coverslip when becoming rounded upon stimulation with cAMP (Alcantara & Monk, 1974). While the model could be modified to agree with each experimentally-observed detail by introducing additional fitting parameters, this would significantly complicate the model and limit its power in predicting phenomena. Since our model predictions do not depend on spike shape but general phenomenology, we chose not to do this here. Nonetheless, our experimental results suggest that these alternative models will share certain basic fundamental features with our FHN-based model, including a core negative feedback loop that gives rise to oscillations and stochasticity.”

Reviewer #3:

I am pleased to see that with their manuscript revision, Sgro and colleagues have addressed most of the concerns raised in the initial reviews. New data and several clarifications throughout the revised manuscript have further strengthened the paper, helping, in particular, to explain pieces of data that had previously seemed to undermine some of the interpretations in the original draft. Overall, the manuscript is clear, well written and the model that is presented is well supported by several validation experiments.

A few suggestions for additional minor changes:

1. Regarding Reviewer 1's second point - the authors could add a brief statement to the result section addressing the issue that although an excitable system is expected to produce very similar spikes in all cells, changes in cell shape may be what causes different cells to have differently shaped spikes upon stimulation with cAMP. Although they now address how they deal with cells that reduce contact with the coverslip in the methods, readers it's a sufficiently important point to be brought up in the description of the results.

We have also added a statement to the text concerning this issue. Specifically, we now state on page 23 paragraph 1, “Finally, we note that the FHN model does not reproduce the experimentally-observed spike shape and that the experimentally-observed spike shape does not always match the average, stereotyped response, likely due to cells that do not remain flat on the surface of the coverslip when becoming rounded upon stimulation with cAMP (Alcantara & Monk, 1974).”

2. P.36, caption for figure 1. The caption only describes 2 of the 3 curves in the panel.

We thank the reviewer for finding this omission and have updated the figure legend to describe all three data traces in the figure.

3. P.25, bottom paragraph. "higher initial average response than those in microfluidic dishes..." - should this be "macrofluidic"?

We thank the reviewer for finding this typo and have corrected the sentence to read "higher initial average response than those in macrofluidic dishes".

4. In multiple figures, there are green dashed vertical lines indicating the start of treatment, but although they are visible on my screen, they did not print (may be too thin?)

We have increased the thickness of both these green dashed vertical lines in figures 1, 3, 4, 6, 7, E1 and the red dashed vertical lines in figure 4.

5. It would be helpful to indicate in each figure caption whether the experiment was performed in the micro- or macro-fluidic setup (which would explain, for example, the three orders of magnitude difference in flow rates in figure 5 vs. figure 7).

All new experiments in this paper are performed in microfluidic devices, except for the experiments shown in Figure E2 comparing the responses of cells in microfluidic devices to macrofluidic dishes. We have added text to each figure caption to indicate whether microfluidic devices or microfluidic dishes were used.

3rd Editorial Decision 08 December 2014

Thank you for sending us your revised manuscript. We are now satisfied with the modifications made and we think that the work is suitable for publication in Molecular Systems Biology.

Before we formally accept the study, we would like to ask you to provide the source data used to generate the figures (in the form of .csv or .txt or .xls files) as Supplementary Datasets accompanying the paper, in order to ensure their long-term archival.

3rd Revision - authors' response 13 December 2014

We have now updated all data files to .xls files as requested.
